# Study on Intervention Mechanism of Yiqi Huayu Jiedu Decoction on ARDS Based on Network Pharmacology

**DOI:** 10.1155/2020/4782470

**Published:** 2020-08-11

**Authors:** Xu Liang, Changyong Luo, Yan Li, Xin Li, Qian Wang, Shujing Zhang, Qingqiao Sun, Yuanhong Ma, Caihua Xiong, Yanpeng Zeng

**Affiliations:** ^1^Beijing University of Chinese Medicine, Beijing, China; ^2^Dongzhimen Hospital of Beijing University of Chinese Medicine, Beijing, China; ^3^College of Life Sciences, Beijing University of Chinese Medicine, Beijing, China; ^4^College of Traditional Chinese Medicine, Beijing University of Chinese Medicine, Beijing, China; ^5^Department of General Medicine, Hebi People's Hospital, Hebi, China

## Abstract

**Background:**

Yiqi Huayu Jiedu (YQHYJD) is a traditional Chinese medicine decoction made up of eight traditional Chinese medicines. Although YQHYJD is effectively used to prevent and treat ARDS/acute lung injury (ALI) in rats, the molecular mechanisms supporting its clinical application remain elusive. The purpose of the current study was to understand its lung protective effects at the molecular level using network pharmacology approach.

**Methods:**

In an ARDS animal model, the beneficial pharmacological activities of YQHYJD were confirmed by reduced lung tissue damage levels observed on drug treated rats versus control group. We then proposed a network analysis to discover the key nodes based on drugs and disease network. Subsequently, we analyzed interaction networks and screened key targets. Using Western blot to detect the expression level of key targets, the intervention effect of changes in expression level of key targets on ARDS was evaluated.

**Results:**

Pathway enrichment analysis of highly ranked genes showed that ErbB pathways were highly related to ARDS. Finally, western blot results showed decreased level of the AKT1 and KRAS/NRAS/HRAS protein in the lung after treatment which confirmed the hypothesis.

**Conclusion:**

In conclusion, our results suggest that YQHYJD can exert lung tissue protective effect against the severe injury through multiple pathways, including the endothelial cells permeability improvement, inflammatory reaction inhibition, edema, and lung tissue hemorrhage reduction.

## 1. Background

Acute respiratory distress syndrome (ARDS) is a common acute critical illness. The main pathophysiological changes are reduced lung volume, reduced lung compliance, and severe ventilation/blood flow imbalance, with a mortality rate of up to 40% [1, 2], and the main treatment strategy is to actively treat the primary disease, mechanical ventilation, oxygen therapy, and restrictive fluid management [3]. Many clinical applications of glucocorticoids, nitric oxide inhalation, pulmonary surfactants, and symptomatic treatments have achieved certain effects in animal experiments but have not achieved satisfactory clinical results and may have more serious clinical side effects [4–6]. Traditional Chinese medicine (TCM) has a unique understanding on the pathogenesis of ARDS. It is believed that the main reason for the occurrence and development of ARDS is that the normal physiological function of the lung is weakened and the pathological factors are increased. Related studies have shown that TCM has obvious effects in reducing ARDS/ALI lung tissue inflammation, reducing mortality, and improving prognosis. TCM compound has multiple targets. Compared with a single blocker, it has better clinical advantages in treating diseases, and has broad application prospects for the prevention and treatment of ARDS [7–9].

As an important intervention due to its significant preventive and therapeutic effects, TCM treatment included novel coronavirus pneumonia diagnosis and treatment plan in China's fight against the epidemic of COVID-19 [10]. Novel coronavirus pneumonia is significantly improved in the treatment of integrated traditional Chinese and Western medicine in improving clinical symptoms and reducing hospitalization time [11]. At the same time, TCM treatment plays an important role in reducing the development of sepsis, ARDS, and patient mortality [12]. In the previous meta-analysis study, we found that the combination of traditional Chinese and Western medicine in the treatment of ARDS has significant advantages in improving the mortality rate and reducing the time of mechanical ventilation compared with simple support treatment [13].

YQHYJD is the clinical experience decoction of Professor Du Huaitang from Dongzhimen Hospital of Beijing University of Chinese medicine. It is composed of 8 traditional Chinese medicines, which are commonly used in the clinical practice of traditional Chinese medicine, namely, *Radix Astragali* (the dried roots of *Astragalus membranaceus (*Fisch*.)* Bunge, also named as Huangqi in Chinese), *Panax notoginseng* (the dried roots of *Panax notoginseng (*Burk.*) F. H. Chen*, also named as Sanqi in Chinese), *Flos Lonicerae* (the dried flowers of *Lonicera japonica* Thunb., also named as Jinyinhua in Chinese), *Radix Scutellariae* (the dried roots of *Scutellaria baicalensis Georgi*, also named as Huangqin in Chinese), *Radix Paeoniae Rubra* (the dried roots of *Paeonia lactiflora* Pall. or *Paeonia veitchii* Lynch*.,* also named as Chishao in Chinese), *Fructus Aurantii Immaturus* (the dried fruits of *Citrus aurantium L*. or *Citrus sinensis* Osbeck*.,* also named as Zhishi in Chinese), *Cortex Mori* (the dried root barks of *Morus alba L*., also named as Sangbaipi in Chinese), *Semen Descurainiae* (the dried seeds of *Lepidium apetalum* Willd. or *Descurainia sophia (*L.) Webb. ex Prantl, also named as Tinglizi in Chinese).

Eight traditional Chinese medicines in YQHYJD are used with reference to the traditional efficacy of TCM in the “Pharmacopoeia of the People's Republic of China” [14]. Pharmacological studies have shown that some of the active ingredients in these traditional Chinese medicines have anti-ALI/ARDS effects, such as *Astragaloside* IV in *Radix Astragali* which can alleviate LPS-induced acute lung injury by inhibiting the activation of NF-*κ*B pathway and the expression of inflammatory genes [15]. *Astragalus polysaccharide* inhibits the expression of ICAM-1 and VCAM-1 in TNF-*α* treated human vascular endothelial cells by blocking NF-*κ*B activation [16]. PNS (*Panax notoginseng saponins*) is very effective in promoting blood circulation to dissipate blood stasis and in improving blood circulation [17]; at the same time, ginsenoside Rb1 in *Panax notoginseng* can reduce the expression of ICAM-1 and ICAM-1, improve the connection between pulmonary microvascular endothelial cells, and reduce lung injury [18]. Baicalein, main component of *Radix Scutellariae*, can prevent LPS-induced acute lung injury in rats [19]. lx-1-mediated endothelial oxidative damage was attenuated by baicalein regulating AMPK/PKC/NADPH oxidase/NF-*κ*B signaling pathway. Therefore, baicalein reduced the formation and dysfunction of SOD-1 ROS induced by NADPH oxidase [20]. *Flos Lonicerae*'s active ingredient quercetin negatively regulates TLR4 signaling induced by lipopolysaccharide through Tollip expression and quercetin and quercitrin protect against cytokine induced injuries in RINm5F *β*-cells via the mitochondrial pathway and NF-*κ*B signaling; these active compounds of *Flos Lonicerae* have been shown to exhibit a variety of modulatory effects on the inflammation process by reducing the induction of proinflammatory cytokines [21, 22]. *Radix Paeoniae Rubra* is able to reduce heat and cool blood, disperse stasis, and relieve pain. It was observed that total paeony glycoside (effective component of *Radix Paeoniae Rubra*) can inhibit apoptosis and oxidative stress by significantly reducing the phosphorylation level of PI3K and Akt and then regulate hemodynamics [23]. Hesperidin, neohesperidin, and neohesperidin dihydrochalcone are the main effective components in *Fructus Aurantii Immaturus;* they have been proved to have the properties of antioxidant [24], anti-inflammatory [25], vasopressive, and antiplatelet [26], which may play a preventive and therapeutic effect in the process of ALI. Research shows that morusin (the effective component of *cortex mori*) treatment led to decreased levels of proinflammatory cytokines such as interleukin, IL-6, and IL-1*β*, and TNF-*α* and increased level of anti-inflammatory IL-10 in mice lung tissue. Morusin relieves mycoplasma pneumonia via the inhibition of the activation of Wnt/*β*-catenin and NF-*κ*B pathways [27]. *Semen Descurainiae* extract administration significantly reduced mucus production, inflammatory cell infiltration into airways, and eosinophil activation by reducing the expression of type 2 cytokines and inhibiting differentiation and activation of Th2 cells [28]. In addition, many effective components in these traditional Chinese medicines have been proved to have the functions of anti-inflammation, antioxidation, reducing cell damage, inhibiting cell apoptosis, and oxidative stress, which play an important role in the treatment of ALI/ARDS.

According to the clinical observation for many years, we found that the series prescription of YQHYJD had a significant effect on the treatment of critical respiratory system diseases [29], and our previous work [30–33] shows that YQHYJD has protective effects on lung injury caused by endotoxin in ARDS rats. The compound can reduce alveolar collapse, reduce inflammatory cell exudation, and at the same time reduce pulmonary edema and improve lung permeability. And it can reduce serum tumor necrosis factor-*α* (TNF-*α*), interleukin-1*β* (IL-1*β*), interleukin-6 (IL-6), interleukin-8 (IL-8), and other proinflammatory factors, reduce NF-*κ*B p50, p65, mitogen-activated protein kinase p38, and other inflammatory proteins expressions, and increase anti-inflammatory factors I*κ*B*α*, interleukin-4 (IL-4), and interleukin-10 (IL-10) expression. At the same time, the group confirmed the intervention effect of the compound on ARDS at the level of proteomics, but the mechanism of action was not clear.

The concept of network pharmacology was firstly systematically elaborated by the British pharmacologist Andrew L. Hopkins in 2007. The “disease-gene-target-drug” interaction network is the research object, and a systematic analysis of the interaction relationship between drugs and the body is conducted to discover new drugs and explore the research methods of drug efficacy and mechanism of action [34]. Using online pharmacology combined with experimental verification, it was found that the therapeutic effect of Dengzhan Xixin Injection on ischemic stroke is mainly through the intervention of NF-kappa B signal, TNF signal, and PI3K-Akt signal, and then it exerts the dual role of anti-inflammatory and antiplatelet effect [35]. In the network pharmacology study of Huanglian Jiedu decoction for the treatment of “internal fire,” it was found that the mechanism of action of this prescription mainly includes anti-OS/NS, anti-inflammatory, and anti-infection, mainly achieved by regulating PI3K-AKT, MAPK, VEGF, and calcium signals conductive pathway [36]. TCM compound has the characteristics of multiple component compatibility and multiple target systems, which is consistent with the research ideas of the integrity and dynamics of network pharmacology. Network pharmacology is emerging in the field of TCM research [37].

**Figure 1:**
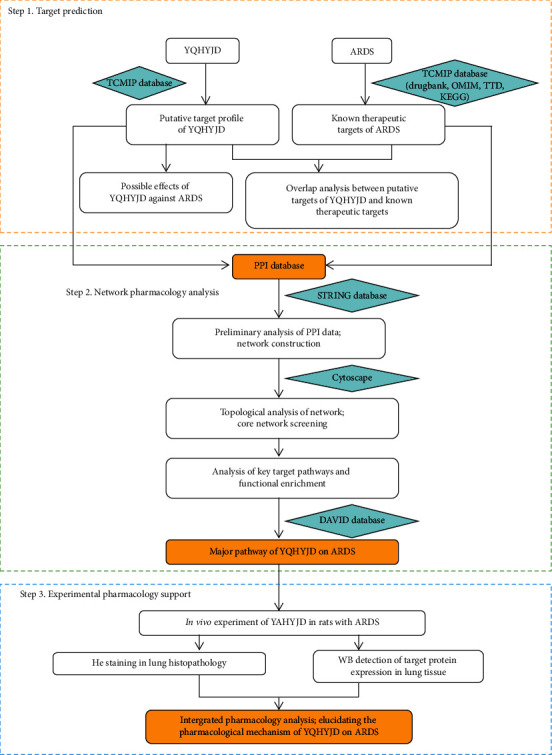
The flowchart of network pharmacology-based strategy for deciphering the mechanisms of YQHYJD acting on ARDS. YQHYJD, Yiqi Huayu Jiedu decoction; TCMIP, integrative pharmacology-based research platform of traditional Chinese medicine; OMIM, Online Mendelian Inheritance in Man; TTD, therapeutic target database; KEGG, Kyoto Encyclopedia of Genes and Genomes; PPI, protein-protein interaction.

In this work, based on the network pharmacology research strategy, we integrate bioinformatics methods such as network construction and analysis, pathways, and target prediction to study the relevant molecular networks of YQHYJD in ARDS rats. Combined with animal experiments, the prediction results are validated and the mechanism of the compound intervention in ARDS is explored. The work in this paper can provide valuable references for the further quality control, product development, and clinical application of the compound.  Figure 1 depicts the flowchart of our study.

## 2. Methods

### 2.1. Materials and Chemicals

LPS *Escherichia coli* and PBS solutions were purchased from Sigma Company in the United States; chloral hydrate, paraformaldehyde, absolute ethanol, xylene, methanol, and so on were purchased from Beijing Chemical Industry Group Co., Ltd. Hematoxylin and eosin dyes were purchased from Beijing Zhongshan Jinqiao Biotechnology Co., Ltd. Liquid paraffin, RIPA lysate, 30% polyacrylamide solution-bisacrylamide, 4 × separation gel buffer, 4 × concentrated gel buffer, 10 × electrophoresis solution, 10 × electrophoresis solution, and so on were purchased from Beijing Solibao Technology Co., Ltd. BCA protein quantitative analysis kit, protease inhibitor (100x), 10% ammonium persulfate solution, TEMED, skimmed milk powder, and TBST were purchased from Beijing Pulailai Gene Technology Co., Ltd. Anti-AKT1 antibody and anti-KRAS/HRAS/NRAS antibody were purchased from Abbot (Shanghai) Trading Co., Ltd. Primary antibody dilutions were purchased from Shanghai Biyuntian Biotechnology Co., Ltd. Goat anti-rabbit IgG/HRP was purchased from Beijing Baierdi Biotechnology Co., Ltd. Chemiluminescence liquid was purchased from GE Company in the United States.

### 2.2. Animals

A total of 30 clean male SD rats were selected, aging 8 weeks old and weighing 190–210 g (Beijing Weitong Lihua Experimental Animal Technology Co., Ltd.). They were fed in the Barrier Laboratory of Experimental Animal Center, Dongzhimen Hospital, Beijing University of Chinese Medicine (6 rats per cage, temperature 22–24°C, humidity 50–70%, free drinking water, and solid particle feed). The experiment was approved by the Experimental Animal Welfare and Ethics Committee of Dongzhimen Hospital of Beijing University of Chinese Medicine. The international experimental pharmacological principles were strict when handling animals.

### 2.3. Source and Preparation of YQHYJD

The composition of YQHYJD used for research was *Radix Astragali* 30 g, *Panax Notoginseng* 10 g, *Flos Lonicerae* 15 g, *Radix Scutellariae* 10 g, fried *Fructus Aurantii Immaturus* 12 g, *Radix Paeoniae Rubra* 15 g, *Semen Descurainiae* 15 g, and *Cortex Mori* 15 g; the medicinal materials used in the research are from Bozhou Yonggang Decoction Pieces Factory Co., Ltd. and the formulation was a TCM extract. Use 10 times the quality of water to decoct the traditional Chinese medicine for 2 times, each time for 30 minutes. The decoction obtained from the two decoctions was mixed, centrifuged, concentrated to 1.22*g* crude drug/ml, and finally stored at 4°C before use, which was produced and provided by Institute of Chinese Materia Medica, Chinese Academy of Chinese Medical Sciences (Batch No.:20170320).

### 2.4. Establishment and Analysis of ARDS-Related Molecular Network of Potential Targets of Chinese Materia Medica in YQHYJD

TCMIP (V1.0) [38] database of Chinese herbal medicines is based on the *Chinese Pharmacopoeia* (2015 edition), which contains more than 400 commonly used Chinese herbal medicines. In the Chinese medicine information setting interface, the 8 Chinese herbs contained in YQHYJD were searched and collected separately. For all chemical components, all drug targets with a similarity score ≥0.8 were selected as potential targets for YQHYJD at the Chinese medicine target prediction interface.

TCMIP disease/symptom target database integrates biological database information such as Drugbank [39], KEGG [40], OMIM [41], TTD [42], and HPO [43]. Through the TCMIP disease target information data retrieval interface, the English abbreviation “ARDS” was searched as a keyword to obtain information about the disease targets currently known to treat ARDS.

### 2.5. Collection of PPI Information

By integrating the above potential drug targets and ARDS disease targets and inputting into the STRING database (V11.0) [44], the PPI network was calculated and generated. Adjusting the network parameters, the “confidence” was used as the confidence of the edges in the network to set the minimum interaction score as 0.9 (highest confidence). The ARDS-related molecular network information of the potential target of Chinese herbs contained in YQHYJD was calculated and saved.

The PPI network data obtained in the above steps were input into Cytoscape software (V 3.5.1) [45] for network visualization and analysis and established “the potential target-ARDS related molecular network of Chinese herbs contained in YQHYJD.” By calculating the PPI network topology characteristic value, the median of degree, closeness centrality, and betweenness centrality as the card values, and the nodes that meet the three card values at the same time were selected as the key network nodes.

The key nodes of the above network were input into the DAVID database (V6.8) [46], and related pathways and biological function enrichment analysis were performed. The target pathways and related target genes were analyzed combined with the literature, and the key targets for the compound intervention in ARDS were screened. Animals' experiments were designed for verification.

### 2.6. *In Vivo* Animal Experiment

The speculated mechanism of YQHYJD in the treatment of ARDS by a network pharmacological method was then verified by polypharmacology using an ARDS model.

### 2.7. Experimental Scheme

A total of 30 SD rats were randomly divided into 5 groups of a normal control group (NC), a model control group (MC), a low-dose group (LDG), a medium-dose group (MDG), and a high-dose group (HDG), with 6 rats in each group. The rats were weighed and recorded, with adaptive feeding for 1 week. Weighing and recording before each experiment. The dosage was calculated according to the adult's weight of 60 kg. The dose calculated by the rats and adults based on a factor of 6.25 times was the Chinese medicine dose. The low was 0.5 times as dose of the medium, and the high was 2 times as dose of the medium. Chinese medicine extract was prepared into 6.1 g/kg/d, 12.2 g/kg/d, and 24.4 g/kg/d (refer to the weight of the crude medicine) Chinese medicine decoction, which were equivalent to clinical equivalent doses of 0.5, 1, and 2 times, respectively. After the end of the adaptive feeding, Chinese medicine was administered to prevent the stomach. The normal group and the model group were given 1 mL/100 g/d of distilled water for 7 consecutive days. Each group had normal drinking water. The Chinese medicine solution was prepared with distilled water, and the medicinal solution and distilled water were stored in a refrigerator at 4°C and heated in a warm water bath before the operation of gastric administration.

Inflammatory ARDS rat model was established by tail vein injection of lipopolysaccharides (LPS). Previous research of the research group proved that the modeling method was stable and effective [30, 47–49]. After gavage for 6  hours on day 7, the rats were weighed and recorded. The model group and each Chinese medicine group were injected with LPS (2 mg/kg body weight) in the tail vein to establish the ARDS model caused by infection. The normal control group was injected with normal saline (2 mg/kg body weight) in the tail vein. Materials were obtained under anesthesia at 16 hours after modeling, and rats were deprived of water for 8 hours before the materials were obtained. Rats were anesthetized by intraperitoneal injection of 1.5% pentobarbital sodium solution (2 ml/kg) and exposed to chest cavity. After sampling, continue to inject appropriate pentobarbital sodium solution until respiratory arrest, and then cervical dislocation, confirm euthanasia. The whole lungs were removed from the level of tracheal cartilage and quickly placed in a cryogenic dish filled with PBS. The sterile equipment was used to take the right lung tissue of the rat under sterile conditions, and placed the upper 1/3 of the lung tissue in a fixed solution (4% paraformaldehyde solution) for pathological examination, and the remaining 2/3 of the lung tissue was stored in a refrigerator at 80°C for western blot to detect the expression level of the target protein.

### 2.8. Observation of Lung Injury

The pathological changes of the lungs, including color, degree of edema, and degree of congestion, were observed with naked eye and recorded with a digital camera. The right lung tissue specimen was taken and paraffin sections were made through fixing, washing, dehydrating, clearing, immersing in wax, and embedding. After staining with hematoxylin and eosin (HE), it was observed under an optical microscope.

Western blot method was used to detect the expression level of potential target protein. Lung tissue was subjected to protein extraction and protein concentration was determined using BCA method. The target protein expression level was determined by SDS-PAGE electrophoresis, membrane transfer, protein-antibody reaction, chemiluminescence, and development.

### 2.9. Statistical Analysis

In this study, SPSS 21.0 software was used for statistical analysis. Each group of data was expressed as mean ± standard deviation $\left(\bar{x}\pm s\right)$. When variances were uniform, one-way analysis of variance was used to test for significance of differences. When variances were uneven, rank sum test was used. *P* < 0.05 was the standard with significant difference. Graphpad Prism 7 software and Adobe Photoshop CS6 version software were used to make data chart.

## 3. Results

### 3.1. Network Pharmacology-Based Analysis

#### 3.1.1. Prediction of Potential Targets of YQHYJD

The TCMIP database of Chinese herbal medicines was used to search the 8 Chinese herbal ingredients contained in YQHYJD, and a total of 369 Chinese herbal ingredients were collected, of which *Radix Astragali, Panax Notoginseng, Flos Lonicerae, Radix Scutellariae, Radix Paeoniae Rubra, Fructus Aurantii Immaturus, Cortex Mori,* and *Semen Descurainiae* were collected with the number of 29, 95, 47, 54, 20, 49, 54, and 21 (Supplementary Figure 1). The predicted target numbers and the common targets among drugs were shown in Tables 1 and 2. The analysis of common target of drugs means the correlation analysis of different Chinese medicines acting on the same target, which means that there is mutual synergy or antagonism between Chinese herbs. The more the common targets are, the stronger the synergy or antagonism will be. A total of 278 potential targets were obtained for the compound (Supplementary Figure 2).

The above-mentioned drug targets were analyzed by gene ontology (GO) and Kyoto Encyclopedia of Genes and Genomes (KEGG) enrichment analysis. It was found that the drug targets involved in biological functions mainly include oxidation-reduction process, mitochondrial electron transport, mitochondrial respiratory chain complex I assembly, membrane depolarization during action potential, aldehyde dehydrogenase (NAD) activity, voltage-gated sodium channel activity, NF-kappaB signaling, and response to oxidative stress (Supplementary Figure 3); drug target participation pathways mainly include oxidative phosphorylation, HIF- 1 signaling pathway, mTOR signaling pathway, apoptosis, VEGF signaling pathway, gap junction, AMPK signaling pathway, NF-kappa B signaling pathway, FoxO signaling pathway, Ras signaling pathway, and MAPK signaling pathway (Supplementary Figure 4).

#### 3.1.2. Network Establishment and Analysis of YQHYJD Intervening in ARDS

**Figure 2:**
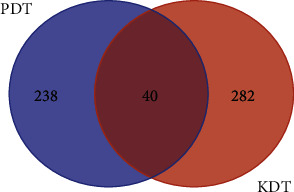
The Venn diagram of the targets both in ARDS targets and YQHYJD targets. PDT, putative drug targets; KDT, known disease targets.

In this study, a total of 322 pieces of target information for ARDS diseases were obtained (Supplementary Figure 5). Integrating drug-related targets and disease-related targets, it was found that there were 40 targets in the 2 types of target profiles (Figure 2). They were NSDHL, CASK, DYRK1A, GCK, ALDH3A2, PIK3R1, AKT1, B4GALT1, HSD17B10, EHHADH, CHUK, MAPK1, YWHAE, PTPN11, GAA, KRAS, PIK3R2, INS, ATP5A1, TUBG1, GNAS, DLD, GCDH, UQCRC2, CPT2, GDI1, HNF1A, PPARG, ABCC9, RPS6KA3, PDHA1, MAPT, P4HB, GRIN2B, ACSL4, ACOX1, VCP, ABCC8, TNFSF11, and PTS. The existence of common targets indicated that there is overlap between the potential drug target and the disease treatment target that has been supported by evidence, further indicating that YQHYJD has sufficient scientific connotation to prevent and treat ARDS.

**Figure 3:**
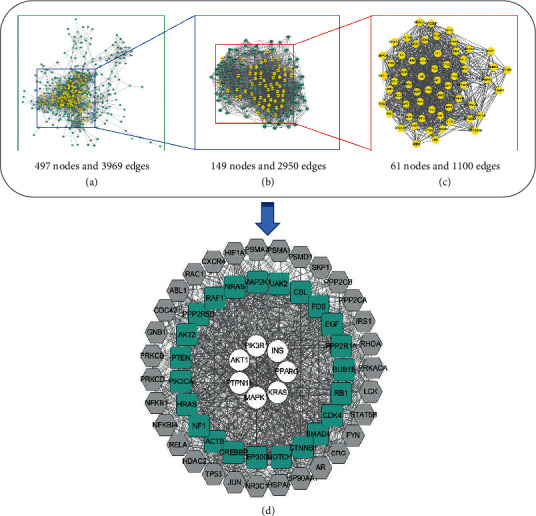
Construction of YQHYJD-ARDS PPI network. (a) Interactive PPI network of YQHYJD and ARDS with 497 nodes and 3969 edges. (b) First central network evaluation with a core subset of 149 nodes and 2950 edges based on the median degree of 12. (c) Second central network evaluation with a core subset of 61 nodes and 1100 edges based on ““Degree” > 35, “betweenness centrality” >0.002, “closeness centrality” >0.56”. (d) Network diagram of 61 core targets. White nodes, green nodes, and gray nodes stand for common targets, known disease targets, and putative drug targets, respectively.

The PPI data calculated by STRING were input into the Cytoscape software to establish the “potential target -ARDS-related molecular network of Chinese medicine contained in YQHYJD.” By analyzing and calculating the network characteristic values, 61 key network nodes were selected (Table 3). After visualization, Figure 3 was obtained.

**Figure 4:**
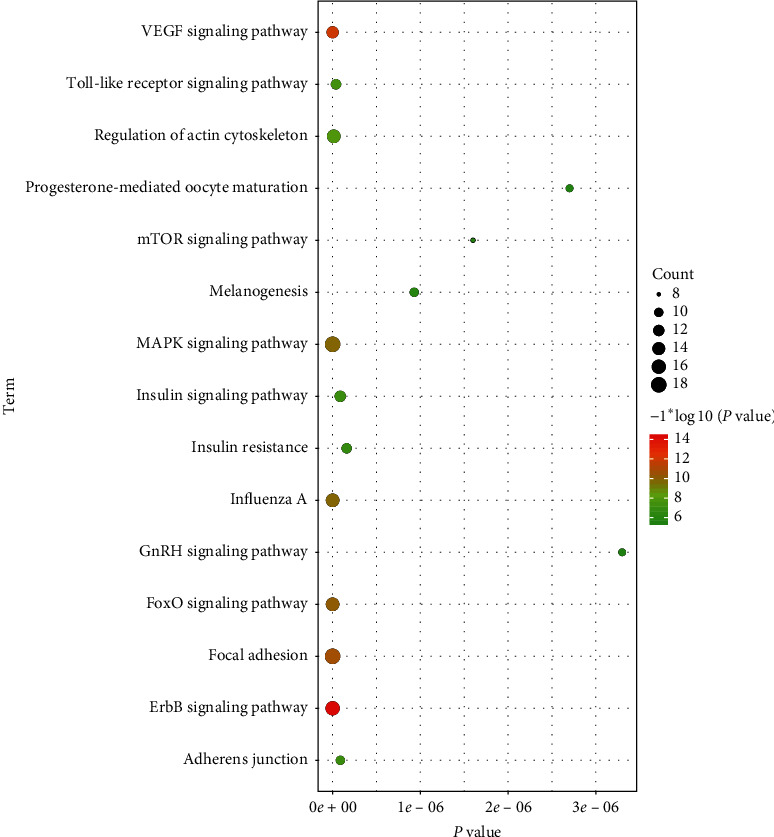
DAVID enrichment analysis of the 61 core targets.

**Figure 5:**
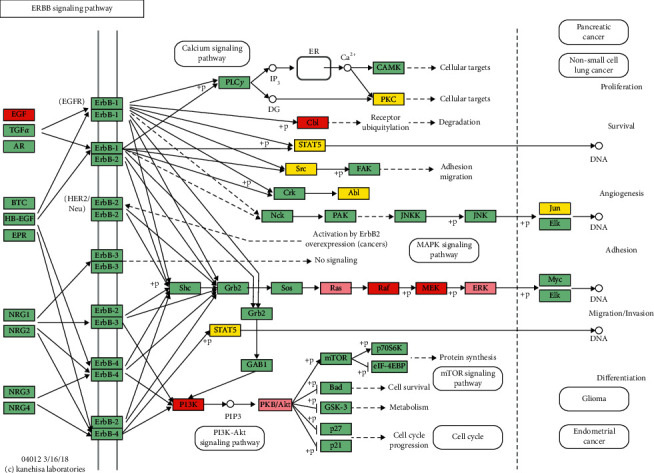
The anti-ARDS pathway of YQHYJD in KEGG. The red nodes represent the known disease targets, the yellow nodes represent the putative drug targets. The pink nodes represent the common targets, and the green nodes represent the targets in the pathway.

The key targets of the above network in the DAVID database were analyzed to obtain the related pathways and biological function information (Figure 4) and sort them by importance (Table 4 only listed entries with *P* value <9.0 × 10^−6^). Key target functions mainly involve ErbB signaling pathway, VEGF signaling pathway, focal adhesion, FoxO signaling pathway, influenza A, MAPK signaling pathway, regulation of actin cytoskeleton, Toll-like receptor signaling pathway, insulin signaling pathway, adherens junction, mTOR signaling pathway, gap junction, Ras protein signal transduction, positive regulation of ERK1 and ERK2 cascade, and so on. The correlation between ErbB signaling pathway with the highest *P* value and key targets was analyzed, and 14 key targets were found to be enriched in ErbB signaling pathway, as shown in Figure 5.

By analyzing the upstream and downstream of the ErbB signaling pathway, it was found that key targets were mainly located on the downstream PI3K-AKT signal axis and MAPK signal axis. Combined with our previous research results, it was believed that YQHYJD might play an interventional role in ARDS by regulating these 2 signal axes. In order to verify the network analysis and prediction results, animal experiments were designed for verification.

### 3.2. Evaluation and Analysis of YQHYJD on ARDS *in Vivo*

#### 3.2.1. Observation and Evaluation of Lung Injury

**Figure 6:**
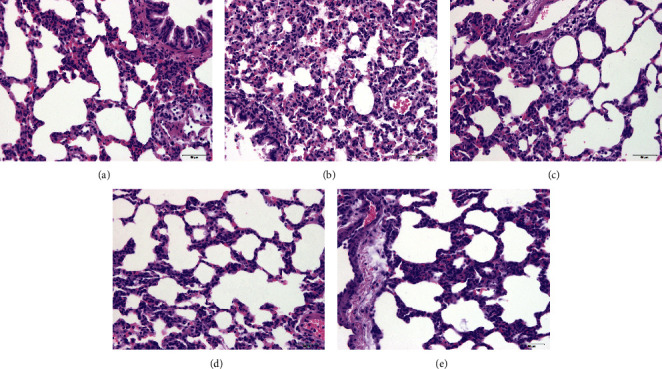
Comparison of pathological morphology of the lung tissue of the rat (×400): (a) NC group, (b) MC group, (c) low-dose group, (d) middle-dose group, and (e) high-dose group.

During the onset of ARDS, the occurrence of explosive inflammatory reactions led to pathological changes in lung tissue damage. Therefore, it is of great significance to reduce inflammation damage and protect lung tissue function in terms of treatment. As shown in Figure 6, after HE staining, microscopic observation showed that the normal group had clear lung tissue structure, complete alveoli, normal alveolar septum, no edema, no exudation of alveolar cavity and lung tissue, and no congestion and inflammatory cell infiltration. In the model group, the lung tissue structure was damaged. Part of the alveolar atrophy collapsed. The alveolar cavity was reduced. The alveolar wall was thickened, and alveolar, interstitial, and bronchi were exuded and tissue edema, inflammatory cell infiltration, and red blood cells were seen in some alveoli and bronchi. Pulmonary capillaries congestion and edema were visible; the degree of lung tissue damage in the low-dose Chinese medicine group was lighter than that in the model group, with a small amount of collapsed alveoli and a small amount of fluid exudation in the alveolar cavity and lung tissue. Some alveolar walls thickened, and alveolar capillaries slightly congested, with edema; the lung tissue of the medium dose group was lighter than the lower dose group, with mild lung tissue edema. No obvious fluid exudation in the alveolar cavity and no alveolar capillary congestion and edema were observed. The degree of lung tissue damage in the compound high-dose group was lighter than that in the model group. A small amount of collapsed alveoli, alveolar, and interstitial edema can be seen, and alveolar capillaries can be seen with slight blood edema.

#### 3.2.2. Effects of YQHYJD on AKT1 and KRAS/HRAS/NRAS Expression in ARDS Rats

**Figure 7:**
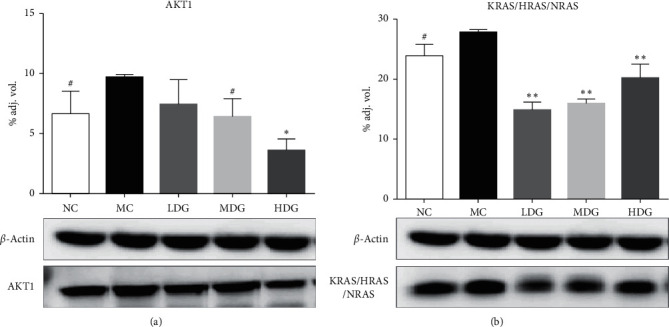
The effect of YQHYJD groups (LDG, 6.1 g/kg; MDG, 12.2 g/kg; HDG, 24.4 g/kg) on the expression of inflammation related protein in the lung of ARDS rats. (a) Expression of AKT1. (b) Expression of KRAS/HRAS/NRAS. All data was presented as mean ± SD (*n* = 5). ^#^*P*, ^*∗*^*P*, and ^*∗∗*^*P* mean the *P* values are less than 0.05, 0.01, and 0.001, respectively, by contrast to model group.

Each dose group can reduce the expression levels of AKT1 and KRAS/HRAS/NRAS in lung tissue of ARDS rats to varying degrees. The compound high-dose group significantly reduced the expression of AKT1 (*P* < 0.01), while there was a dose-dependent inhibition of AKT1 overexpression; each dose group of the compound significantly reduced the expression of KRAS/HRAS/NRAS (*P* < 0.001) (Figure 7).

## 4. Discussion

ARDS is a clinically critical illness characterized by progressive respiratory distress, refractory hypoxemia, and nonuniform exudation of pulmonary imaging [1]. At present, there are many opinions on ARDS that are based on experiments. Most of the opinions based on experimental evidence believe that the explosive inflammatory response caused by various pathogenic factors in the course of ARDS is an important link leading to severe exudation and even bleeding of lung tissue, which leads to severe impairment or loss of lung function [50, 51]. ARDS has a high clinical mortality rate, and finding effective treatment options has become the focus of current research. The Yiqi Huayu Jiedu decoction selected in this study has been proven in the basic research conducted by the research group in the past to effectively reduce the degree of lung tissue damage in ARDS rats and reduce the expression levels of proinflammatory factors and inflammation-related proteins. Our purpose in this study is to systematically analyze and predict the target of the compound intervention in ARDS and to verify it in combination with *in vivo* experiments, to provide a reliable basis for the further research of the compound.

According to the enrichment analysis of the core target pathways of the ARDS network that YQHYJD interferes, the core target pathways include ErbB signaling pathway, VEGF signaling pathway, focal adhesion, FoxO signaling pathway, influenza A, MAPK signaling pathway, Toll-like receptor signaling pathway, insulin signaling pathway, adherens junction, mTOR signaling pathway, gap junction, Ras protein signal transduction, and positive regulation of ERK1 and ERK2 cascade (Table 4). These interactions are related to the pathological mechanism of ARDS. The decrease of VEGF in the lung injury model of cigarette exposure plus LPS inhalation is related to the mechanism that induces ARDS due to increased alveolar capillary permeability, exacerbation of inflammation, epithelial damage, and endothelial dysfunction [52]. The regulation of VEGF signaling pathway is also related to complications such as ALI/ARDS due to lobectomy [53]. Focal adhesions in ARDS and other diseases, through the process of intercellular dynamic signal transduction, cause increased endothelial permeability and inflammatory reactions such as edema [54]. FoxO signaling pathway is involved in the pathological process of pulmonary vascular endothelial cells that reduce tight junctions in ARDS, leading to exudative edema [55]. In the ARDS model caused by influenza, macrophage activation can induce direct apoptosis of alveolar epithelial cells [56]. C3G can play an important anti-inflammatory and antioxidant role by inhibiting the NF-*κ*B pathway and MAPK pathway and improve LPS-induced ARDS/ALI [57]. In ARDS, pro-mRNA splicing of TLR signaling genes has been associated with proinflammatory changes [58]. Insulin stimulates alveolar fluid clearance and reduces ALI pulmonary edema by upregulating the expression of *α*-ENaC, *β*-ENaC, and *γ*-ENaC on the surface of alveolar cells [59]. The mTOR signaling pathway is involved in protecting the endothelial cell barrier, improving vascular endothelial permeability, and interfering with ARDS progression [60]. Gap junction plays an important role in the permeability of the alveolar capillary barrier. Decreasing connexins such as VE-cadherin and Caluin 5 will lead to increased permeability of the ALI/ARDS vascular barrier, which in turn will promote edema formation and respiratory failure [61]. Blocking Ras-associated protein Rab10 can lead to reduced TLR4 expression, reduced production of inflammatory cytokines and interferons, and reduced lung injury [62]. ERK1/2 inhibitors can significantly reduce lung injury in A779 pretreated ACE2 overexpressing rats, thereby reducing LPS-induced ARDS [63]. According to the enrichment results, YQHYJD mainly regulates inflammatory response, vascular endothelial barrier function, and coagulation and fibrinolytic function when intervening in ARDS.

Based on the above analysis, YQHYJD can interfere with ARDS by targeting different genes through multiple signaling pathways. Therefore, we evaluated the predictions through *in vivo* studies. As shown in Figure 6, during the course of ARDS, the compound can protect lung tissues to varying degrees. Each dose group can effectively enhance the ability of rats to resist endotoxin attack. The pathological morphology of lung tissue of ARDS rats in each dose group is better than the model group. There are varying degrees of relief. As shown in Figure 7, the compound middle-dose group can effectively reduce the expression of AKT1, while the high-dose group has a more significant downregulation level. At the same time, the compound has a tendency to inhibit AKT1 overexpression in a dose-dependent manner. Each compound group can significantly reduce KRAS/HRAS/NRAS expression level.

The full name of AKT1 is RAC-alpha serine/threonine-protein kinase, which is involved in regulating cell metabolism, proliferation, cell survival, growth, and angiogenesis through serine and/or threonine phosphorylation of a series of downstream substrates. It is an important downstream signal protein of the PI3K-AKT pathway [64]. AKT1 and its involved PI3K-AKT signaling pathway, mTOR signaling pathway, and VEGF signaling pathway play important roles in the pathological process of regulating ALI/ARDS pulmonary vascular endothelial permeability [65]. In the experiment of edaravone intervention for lung injury, the downregulation of PI3K/AKT pathway protein can reduce the peroxidation reaction and DNA damage, and it can improve the lung injury *in vivo* and *in vitro* experiments [66]. In the experiments of *Staphylococcus aureus*-induced lung injury, the increase in p-AKT expression in the PI3K/AKT cascade was positively correlated with the degree of cell damage [67]. Ligustrazine can improve inflammation and fibrosis in lung tissue of ALIrats, inhibit PI3K/AKT/mTOR pathway activation, reduce p-PI3K/PI3K, p-AKT/AKT, and p-mTOR/mTOR ratios, and reduce LPS-induced ALI[68]. As shown in Figure 5, AKT1 is an important functional protein on the PI3K/AKT signaling pathway and is closely related to the downstream mTOR signaling pathway and VEGF signaling pathway that regulate vascular endothelial function. Therefore, we believe that the role of YQHYJD which reduces lung tissue edema in ARDS rats may be related to reducing AKT1 expression, affecting downstream signaling pathways, and then regulating vascular endothelial permeability.

KRAS, HRAS, and NRAS are different structural monomers of GTPase Ras protein, and they are activation proteins of MAPK transduction pathway. Studies have found that inhibiting the MAPK cascade can reduce lung tissue damage, and its mechanism may be related to inhibiting oxidative stress and apoptosis and blocking inflammatory responses [69–71]. Blocking the phosphorylation of JNK and p38 and inhibiting the MAPK signal can reduce lung permeability and reduce ALI caused by sepsis [72]. The blockage of p38 MAPK signaling pathway caused macrophages to change from inflammatory apoptosis to noninflammatory apoptosis and alleviated the excessive inflammatory response in ALI mice [73]. Studies have found that Xuebijing Injection can alleviate the effects of paraquat by downregulating the p38MAPK-NF-*κ*B inflammatory signaling pathway and blocking the expression of p-p38MAPK, NF-*κ*B65, HIF-1*α*, p-I*κ*B-*α*, and TGF-*β*1 ALI [74]. At the same time, the NF-kappaB signaling pathway is involved in the pathogenesis of ARDS alveolar hypercoagulability and fibrinolysis inhibition [75]. We also found in previous work that YQHYJD can inhibit inflammation-related proteins such as NF-*κ*Bp50, MAPK P38, p65, and the expression of TNF-*α*, IL-1*β*, IL-6, IL-8, and other proinflammatory factors, which plays a protective role in the lung tissue of ARDS rats [30–33]. As shown in Figure 5, Ras protein (KRAS/HRAS/NRAS) is located upstream of the MAPK signaling pathway. Inhibiting this site protein can block the MAPK cascade and affect downstream NF-*κ*B signaling pathway activation, thereby reducing the release of inflammatory proteins and inflammatory factors, which is in line with the results of our previous research. Therefore, we believe that YQHYJD might block the MAPK signaling pathway by inhibiting the expression of KRAS/HRAS/NRAS, thereby reducing lung tissue inflammation.

In this study, each dose group of YQHYJD downregulated the expressions of PI3K-AKT pathway downstream protein AKT1 and MAPK pathway activating protein KRAS/NRAS/HRAS to different extents, which played a role in the intervention of ARDS. The predictions are consistent.

## 5. Conclusion

In short, a systematic study of YQHYJD was conducted through predictive analysis and *in vivo* experiments. The experimental results are consistent with those predicted by network analysis. This study not only evaluated the anti-inflammatory effects of YQHYJD in intervening ARDS, but also proposed that YQHYJD regulates vascular endothelial function and anticoagulant function, which deserves further study.

## Figures and Tables

**Table 1:** Basic information of TCM composition target of YQHYJD.

Chinese Pinyin name	Latin name	Number of components	Number of predicted targets
Huang-qi	*Radix Astragali*	29	412
San-qi	*Panax Notoginseng*	95	239
Jin-yin-hua	*Flos Lonicerae*	47	209
Huang-qin	*Radix Scutellariae*	54	242
Chi-shao	*Radix Paeoniae Rubra*	20	193
Zhi-shi	*Fructus Aurantii Immaturus*	49	131
Sang-Bai-pi	*Cortex Mori*	54	148
Ting-li-zi	*Semen Descurainiae*	21	178
Sum	—	369	1752

**Table 2:** The common target among the components of YQHYJD.

Drugs	*Panax Notoginseng* (239)	*Cortex Mori* (148)	*Radix Astragali* (412)	*Radix Scutellariae* (242)	*Fructus Aurantii Immaturus* (131)	*Radix Paeoniae Rubra* (193)	*Flos Lonicerae* (209)	*Semen Descurainiae* (178)
*Panax Notoginseng* (239)	—	49	150	137	35	71	106	91
*Cortex Mori* (148)	49	—	118	122	106	103	122	106
*Radix Astragali* (412)	150	118	—	216	104	146	167	161
*Radix Scutellariae* (242)	137	122	216	—	105	150	158	159
*Fructus Aurantii Immaturus* (131)	35	106	104	105	—	110	118	105
*Radix Paeoniae Rubra* (193)	71	103	146	150	110	—	145	108
*Flos Lonicerae* (209)	106	122	167	158	118	145	—	114
*Semen Descurainiae* (178)	91	106	161	159	105	108	114	—

**Table 3:** Information on 61 key hub targets.

Uniprot ID	Gene symbol	Target type	Degree	Betweenness	Closeness
P05771	PRKCB	Putative drug target	35	0.00264685	0.56704981
P25786	PSMA1	Putative drug target	36	0.00300257	0.5648855
P06400	RB1	Known disease target	37	0.00253187	0.57142857
Q92769	HDAC2	Putative drug target	38	0.00336775	0.56923077
P63208	SKP1	Putative drug target	38	0.00245534	0.57142857
Q99460	PSMD1	Putative drug target	39	0.00248828	0.57364341
O60566	BUB1B	Known disease target	41	0.00549867	0.56704981
P62873	GNB1	Putative drug target	41	0.00718391	0.56923077
P22681	CBL	Known disease target	42	0.00342413	0.578125
P25963	NFKBIA	Putative drug target	42	0.00433339	0.578125
P04150	NR3C1	Putative drug target	43	0.00468953	0.58498024
P51692	STAT5B	Putative drug target	43	0.00325874	0.58039216
O14818	PSMA7	Putative drug target	43	0.00316088	0.58039216
Q05655	PRKCD	Putative drug target	44	0.00449992	0.58730159
P31751	AKT2	Known disease target	44	0.00270419	0.58730159
P61073	CXCR4	Putative drug target	45	0.00558369	0.58498024
P37231	PPARG	Common target	45	0.00371233	0.58498024
Q13485	SMAD4	Known disease target	47	0.00371397	0.58964143
Q92793	CREBBP	Known disease target	47	0.00699241	0.592
P60709	ACTB	Known disease target	48	0.00650353	0.59677419
P63000	RAC1	Putative drug target	48	0.00516601	0.59677419
Q14738	PPP2R5D	Known disease target	49	0.00893634	0.59677419
P35570	IRS1	Putative drug target	49	0.00452074	0.59919028
P17612	PRKACA	Putative drug target	50	0.01349606	0.60162602
P11802	CDK4	Known disease target	50	0.00628716	0.60162602
P11142	HSPA8	Putative drug target	50	0.01720136	0.60162602
P06239	LCK	Putative drug target	53	0.00312328	0.6090535
P00519	ABL1	Putative drug target	53	0.00590445	0.60655738
Q16665	HIF1A	Putative drug target	53	0.00718986	0.6090535
P06241	FYN	Putative drug target	53	0.00504871	0.6090535
P10275	AR	Putative drug target	54	0.0067063	0.61157025
P21359	NF1	Known disease target	54	0.00762371	0.6090535
P04049	RAF1	Known disease target	55	0.00496974	0.61410788
Q06124	PTPN11	Common target	56	0.00363149	0.61666667
Q02750	MAP2K1	Known disease target	57	0.0043254	0.61924686
P62714	PPP2CB	Putative drug target	57	0.01088017	0.61924686
P19838	NFKB1	Putative drug target	57	0.00880836	0.61666667
P61586	RHOA	Putative drug target	58	0.00571048	0.62184874
P01100	FOS	Known disease target	58	0.00710084	0.61924686
Q09472	EP300	Known disease target	59	0.0174571	0.62447257
O60674	JAK2	Known disease target	60	0.00538805	0.62711864
P01111	NRAS	Known disease target	62	0.00549342	0.63247863
P46531	NOTCH1	Known disease target	63	0.00726887	0.63247863
P27986	PIK3R1	Common target	65	0.00815513	0.64069264
Q04206	RELA	Putative drug target	65	0.01465454	0.64069264
P60953	CDC42	Putative drug target	69	0.01943588	0.65198238
P05412	JUN	Putative drug target	69	0.01511756	0.65198238
P30153	PPP2R1A	Known disease target	71	0.02557709	0.65777778
P42336	PIK3CA	Known disease target	71	0.01292212	0.65777778
P67775	PPP2CA	Putative drug target	73	0.0246169	0.66367713
P01133	EGF	Known disease target	77	0.01462345	0.67579909
P01308	INS	Common target	77	0.0175801	0.67579909
P07900	HSP90AA1	Putative drug target	77	0.02388431	0.67579909
P01116	KRAS	Common target	81	0.01319493	0.68837209
P12931	SRC	Putative drug target	85	0.01745904	0.7014218
P28482	MAPK1	Common target	86	0.02512912	0.7047619
P60484	PTEN	Known disease target	87	0.0220011	0.70813397
P01112	HRAS	Known disease target	87	0.01721804	0.70813397
P35222	CTNNB1	Known disease target	91	0.02966048	0.72195122
P31749	AKT1	Common target	95	0.03102752	0.73631841
P04637	TP53	Putative drug target	105	0.05675909	0.77486911

**Table 4:** Information on 15 pathways.

Term ID	Pathway name	Count	*P* value
hsa04012	ErbB signaling pathway	16	5.10 × 10^−15^
hsa04370	VEGF signaling pathway	13	1.40 × 10^−12^
hsa04510	Focal adhesion	18	2.00 × 10^−11^
hsa04068	FoxO signaling pathway	15	7.20 × 10^−11^
hsa05164	Influenza A	15	1.80 × 10^−10^
hsa04010	MAPK signaling pathway	18	1.80 × 10^−10^
hsa04810	Regulation of actin cytoskeleton	15	1.30 × 10^−8^
hsa04620	Toll-like receptor signaling pathway	11	3.90 × 10^−8^
hsa04910	Insulin signaling pathway	12	8.70 × 10^−8^
hsa04520	Adherens junction	10	8.80 × 10^−8^
hsa04931	Insulin resistance	11	1.60 × 10^−7^
hsa04150	mTOR signaling pathway	8	1.60 × 10^−6^
hsa04540	Gap junction	9	4.00 × 10^−6^
GOTERM_BP_DIRECT	Ras protein signal transduction	5	7.30 × 10^−6^
GOTERM_BP_DIRECT	Positive regulation of ERK1 and ERK2 cascade	7	9.00 × 10^−6^

## Data Availability

The dataset generated during the present study is available upon reasonable request to the corresponding author.

## Abbreviations

YQHYJD: Yiqi Huayu Jiedu
ARDS: Acute respiratory distress syndrome
ALI: Acute lung injury
TCM: Traditional Chinese medicine
NF-*κ*B: Nuclear factor kappa-B
TNF-*α*: Tumor necrosis factor-*α*
LPS: Lipopolysaccharide
IL: Interleukin
TCMIP: Integrative pharmacology-based research platform of traditional Chinese medicine
OMIM: Online Mendelian Inheritance in Man
TTD: Therapeutic target database
HPO: Human phenotype ontology
PPI: Protein-protein interaction
GO: Gene ontology
KEGG: Kyoto Encyclopedia of Genes and Genomes
DAVID: Database for annotation, visualization, and integrated discovery
NC: Normal control
MC: Model control
LDG: Low-dose group
MDG: Medium-dose group
HDG: High-dose group.

## Supplementary Materials

Supplementary Materials There are 5 supplementary figures in the submitted supplementary materials. Supplementary Figure 1: Yiqi Huayu Jiedu decoction ingredients, including Radix Astragali ingredients, Panax Notoginseng ingredients, Flos Lonicerae ingredients, Radix Scutellariae ingredients, Radix Paeoniae Rubra ingredients, Fructus Aurantii Immaturus ingredients, Cortex Mori ingredients, and Semen Descurainiae ingredients. Supplementary Figure 2: potential target information of Yiqi Huayu Jiedu decoction. Supplementary Figure 3: GO enrichment analysis of drug prediction target. Supplementary Figure 4: KEGG enrichment analysis of drug prediction target. Supplementary Figure 5: the target information for ARDS diseases. A graphical abstract is also provided.
