# Supplementary material for: Study on Intervention Mechanism of Yiqi Huayu Jiedu Decoction on ARDS Based on Network Pharmacology
Source: Evid Based Complement Alternat Med. 2020 Aug 11;2020:4782470. doi: 10.1155/2020/4782470 (PMC7439204; doi:10.1155/2020/4782470)
Supplement: Supplementary Materials — There are 5 supplementary figures in the submitted supplementary materials. Supplementary Figure 1: Yiqi Huayu Jiedu decoction ingredients, including Radix Astragali ingredients, Panax Notoginseng ingredients, Flos Lonicerae ingredients, Radix Scutellariae ingredients, Radix Paeoniae Rubra ingredients, Fructus Aurantii Immaturus ingredients, Cortex Mori ingredients, and Semen Descurainiae ingredients. Supplementary Figure 2: potential target information of Yiqi Huayu Jiedu decoction. Supplementary Figure 3: GO enrichment analysis of drug prediction target. Supplementary Figure 4: KEGG enrichment analysis of drug prediction target. Supplementary Figure 5: the target information for ARDS diseases. A graphical abstract is also provided. [file 4782470.f1.zip › Graphical Abstract.pdf]

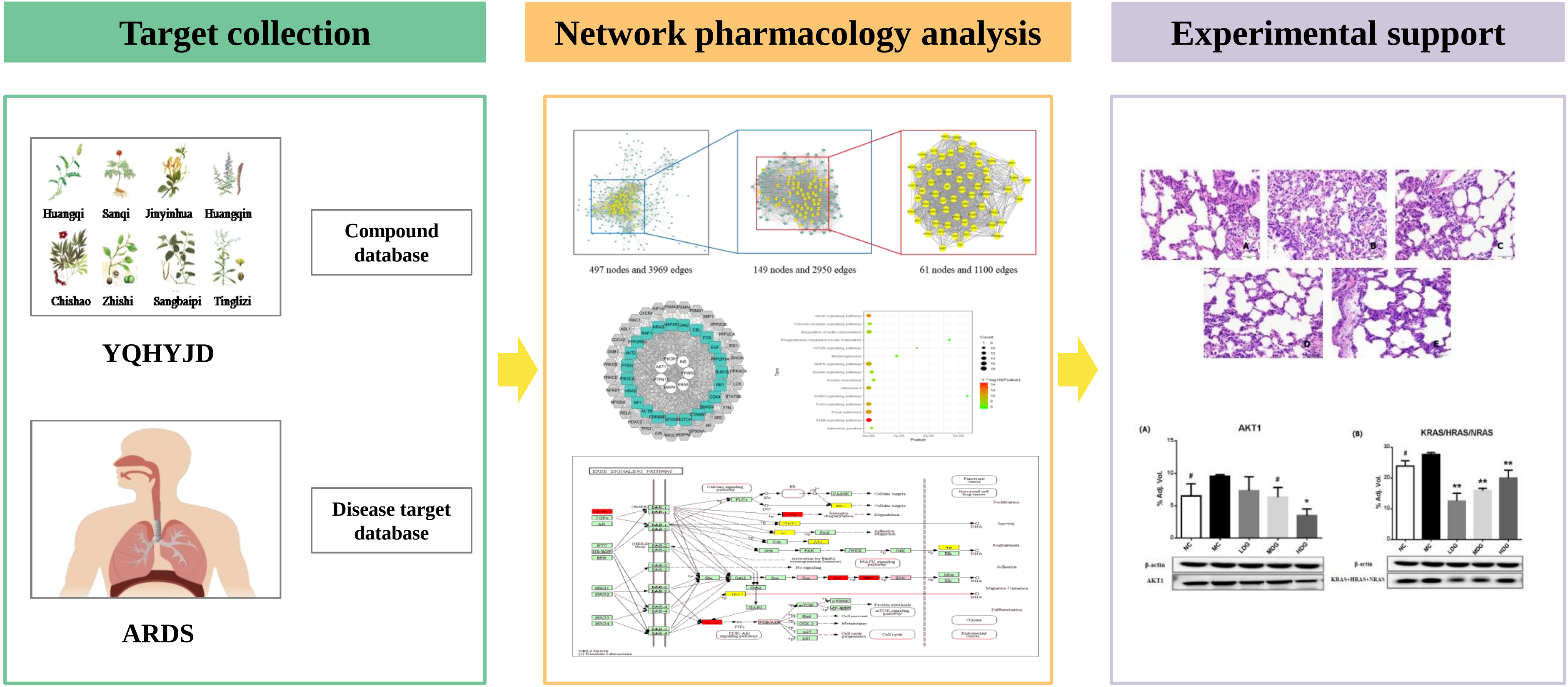

Target collection
Network pharmacology analysis
Experimental support
Huangqi
Sanqi
Jinyinhua
Huangqin
Chishao
Zhishi
Sangbaipi
Tinglizi
Compound database
YQHYJD
Disease target database
ARDS
497 nodes and 3969 edges
149 nodes and 2950 edges
61 nodes and 1100 edges
AKT1
KRAS/HRAS/NRAS
