# Supplementary material for: Study on Intervention Mechanism of Yiqi Huayu Jiedu Decoction on ARDS Based on Network Pharmacology
Source: Evid Based Complement Alternat Med. 2020 Aug 11;2020:4782470. doi: 10.1155/2020/4782470 (PMC7439204; doi:10.1155/2020/4782470)
Supplement: Supplementary Materials — There are 5 supplementary figures in the submitted supplementary materials. Supplementary Figure 1: Yiqi Huayu Jiedu decoction ingredients, including Radix Astragali ingredients, Panax Notoginseng ingredients, Flos Lonicerae ingredients, Radix Scutellariae ingredients, Radix Paeoniae Rubra ingredients, Fructus Aurantii Immaturus ingredients, Cortex Mori ingredients, and Semen Descurainiae ingredients. Supplementary Figure 2: potential target information of Yiqi Huayu Jiedu decoction. Supplementary Figure 3: GO enrichment analysis of drug prediction target. Supplementary Figure 4: KEGG enrichment analysis of drug prediction target. Supplementary Figure 5: the target information for ARDS diseases. A graphical abstract is also provided. [file 4782470.f1.zip › Figures.doc]

**Supplementary Figure 1**

**Yiqi Huayu Jiedu decoction** **ingredients**

***Radix Astragali*** ingredients

| **compound number** | **name** | **molecular formula** |
| --- | --- | --- |
| TCMIP100084 | β-sitosterol | C29H50O |
| TCMIP100249 | Folinic acid | C21H25N7O7 |
| TCMIP100276 | Hexadecanoic acid | C16H32O2 |
| TCMIP100350 | sucrose | C12H22O11 |
| TCMIP100809 | Choline | C5H14NO |
| TCMIP101503 | Folic acid | C19H19N7O6 |
| TCMIP102495 | Kumatakenin | C17H14O6 |
| TCMIP102712 | Βetaine | C5H11NO2 |
| TCMIP103071 | (−)-Medicarpin | C16H14O4 |
| TCMIP103193 | Acetyl astragaloside I | C47H74O17 |
| TCMIP103194 | Astragaloside I | C45H72O16 |
| TCMIP103195 | Astragaloside II | C43H70O15 |
| TCMIP103196 | Astragaloside III | C41H68O14 |
| TCMIP103197 | Astragaloside IV | C41H68O14 |
| TCMIP103198 | Astragaloside V | C47H78O19 |
| TCMIP103199 | Astragaloside VI | C47H78O19 |
| TCMIP103200 | Astragaloside VII | C47H78O19 |
| TCMIP103201 | Astragaloside VIII | C47H76O17 |
| TCMIP103202 | Astramembrannin I | C41H68O14 |
| TCMIP103203 | Astramembrannin II | C35H58O9 |
| TCMIP103204 | Canavanine | C5H12N4O3 |
| TCMIP103205 | Cycloastragenol | C30H50O5 |
| TCMIP103206 | 9,10-Dimethoxy-pterocarpane-3-O-β-D-glucoside | C23H28O10 |
| TCMIP103207 | Glucuronic acid | C6H10O7 |
| TCMIP103208 | 2'-Hydroxy-3',4'-dimethoxy-isoflavane-7-O-β-D-glucoside | C23H32O10 |
| TCMIP103209 | Isoastragaloside I | C45H72O16 |
| TCMIP103210 | Isoastragaloside II | C43H70O15 |
| TCMIP103211 | L-Selenocystathionine | C7H14N2O4Se |
| TCMIP103212 | Astrasieversianin IX | C48H78O18 |

***Panax Notoginseng* ingredients**

| **compound number** | **name** | **molecular formula** |
| --- | --- | --- |
| TCMIP104948 | 20-O-β-D-Xylopyranosyl(1-6)-β-D-glucopyranosyl-20(S)-protopanaxadiol | C41H70O12 |
| TCMIP104947 | 2,2,2-Triethoxyl-ethanol | C8H18O4 |
| TCMIP104946 | Tridecene | C13H26 |
| TCMIP104945 | 1,1,5,5-Tetramethyl-4-methano-2,3,4,6,7,10-hexahydronaphthalene | C15H24 |
| TCMIP104944 | Sanchinoside B1 | C36H62O9 |
| TCMIP104943 | Quinquenoside V | C60H102O28 |
| TCMIP104942 | Quinquenoside IV | C54H90O24 |
| TCMIP104941 | Quinquenoside III | C50H84O19 |
| TCMIP104940 | Quinquenoside II | C62H104O24 |
| TCMIP104939 | Quinquenoside I | C52H86O19 |
| TCMIP104938 | Panaxynol | C17H24O |
| TCMIP104937 | Notoginsenoside R6 | C48H82O19 |
| TCMIP104936 | Notoginsenoside R3 | C48H82O19 |
| TCMIP104935 | Notoginsenoside R2 | C41H70O13 |
| TCMIP104934 | Notoginsenoside N | C48H82O19 |
| TCMIP104933 | Notoginsenoside M | C48H82O19 |
| TCMIP104932 | Notoginsenoside L | C53H90O22 |
| TCMIP104931 | Notoginsenoside K | C54H92O25 |
| TCMIP104930 | Notoginsenoside J | C42H74O16 |
| TCMIP104929 | Notoginsenoside I | C54H92O22 |
| TCMIP104928 | Notoginsenoside H | C47H80O19 |
| TCMIP104927 | Notoginsenoside G | C48H80O19 |
| TCMIP104926 | Notoginsenoside D | C64H108O31 |
| TCMIP104925 | Notoginsenoside C | C54H92O25 |
| TCMIP104924 | Notoginsenoside B | C54H90O24 |
| TCMIP104923 | Notoginsenoside A | C54H92O24 |
| TCMIP104922 | Methyl pyroglutamate | C6H9NO3 |
| TCMIP104921 | 1-Methyl-4-isoallyl-cyclohexane | C10H18 |
| TCMIP104920 | Isobutyl pyroglutamate | C9H15NO3 |
| TCMIP104919 | 6-O-β-D-Glucopyranosyl-20-O-β-D-glucopyranosyl-3β,6α,12β,20(S),25-pentahydroxydammar-23-ene | C42H72O15 |
| TCMIP104918 | 3-O-[β-D-Glucopyranosyl(1-6)-β-D-glucopyranosyl]-20-O-β-D-glucopyranosyl-3β,12β,20(S)-trihydroxydammar-24-ene | C48H82O18 |
| TCMIP104917 | 3β-O-β-D-Glucopyranosyl-20-O-[α-L-arabinopyranosyl(1-2)-β-D-glucopyranosyl]3β,12β,20(S)-trihydroxydammar-24-ene | C47H80O17 |
| TCMIP104916 | Ethyl pyroglutamate | C7H11NO3 |
| TCMIP104915 | 3-Ene-nonanone-2 | C9H16O |
| TCMIP104914 | Ditertbutyl phthalate | C16H22O4 |
| TCMIP104913 | α,α-Dimethylbenzene methanol | C9H12O |
| TCMIP104912 | 2,8-Dimethyl-5-acetyl-bicyclo[5,3,0] decadiene-1,8 | C14H20O |
| TCMIP104911 | Diisocapryl phthalate | C24H38O4 |
| TCMIP104910 | Dicapryl phthalate | C24H38O4 |
| TCMIP104909 | cyperene | C15H24 |
| TCMIP104908 | Cyclo-(Phe-Val) | C14H18N2O2 |
| TCMIP104907 | Cyclo-(Phe-Tyr) | C18H18N2O3 |
| TCMIP104906 | Cyclo-(Phe-Ala) | C12H14N2O2 |
| TCMIP104905 | Cyclo-(Leu-Val) | C11H20N2O2 |
| TCMIP104904 | Cyclo-(Leu-Tyr) | C15H20N2O3 |
| TCMIP104903 | Cyclo-(Leu-Thr) | C10H18N2O3 |
| TCMIP104902 | Cyclo-(Leu-Ser) | C9H16N2O3 |
| TCMIP104901 | Cyclo-(Leu-Ile) | C12H22N2O2 |
| TCMIP104900 | Cyclo-(Leu-Ala) | C9H16N2O2 |
| TCMIP104899 | Cyclo-(Ile-Val) | C11H20N2O2 |
| TCMIP104898 | Cyclo-(Ile-Ala) | C9H16N2O2 |
| TCMIP104897 | Cyclododecanone | C12H22O |
| TCMIP104896 | n-Butyl pyroglutamate | C9H15NO3 |
| TCMIP104895 | L-α-Amino-β-oxalylaminopropionic acid | C5H8N2O5 |
| TCMIP104894 | Acetophenone | C8H8O |
| TCMIP104789 | Tetradecane | C14H30 |
| TCMIP104772 | Panaxytriol | C17H26O3 |
| TCMIP104771 | Panaxydol | C17H24O2 |
| TCMIP104759 | Notoginsenoside R4 | C59H100O27 |
| TCMIP104758 | Notoginsenoside R1 | C47H80O18 |
| TCMIP104754 | α-Muurolene | C15H24 |
| TCMIP104729 | 20(S)-Ginsenoside Rh1 | C36H62O9 |
| TCMIP104725 | Ginsenoside Rg1 | C42H72O14 |
| TCMIP104721 | Ginsenoside Rc | C53H90O22 |
| TCMIP104718 | Ginsenoside Rb1 | C54H92O23 |
| TCMIP104701 | 2,6-Ditertbutyl-4-methyl phenol | C15H24O |
| TCMIP104694 | δ-cadinol / δ-cadinene | C15H24 |
| TCMIP103418 | Cyclo-(Pro-Val) | C10H16N2O2 |
| TCMIP103417 | Cyclo-(Ala-Val) | C8H14N2O2 |
| TCMIP103416 | Cyclo-(Ala-Pro) | C8H12N2O2 |
| TCMIP102266 | Cuparene | C15H22 |
| TCMIP102178 | α-Copaene | C15H24 |
| TCMIP102128 | Eicosane | C20H42 |
| TCMIP102016 | n-Pentadecane | C15H32 |
| TCMIP102002 | Heptadecane | C17H36 |
| TCMIP102001 | Heneicosanoic acid | C21H42O2 |
| TCMIP102000 | Heneicosane | C21H44 |
| TCMIP101996 | Docosane | C22H46 |
| TCMIP101507 | Methyl hexadecanate | C17H34O2 |
| TCMIP101173 | Nonanoic acid | C9H18O2 |
| TCMIP101160 | Enanthic acid | C7H14O2 |
| TCMIP101099 | δ-Guaiene | C15H24 |
| TCMIP101098 | α-Guaiene | C15H24 |
| TCMIP101026 | β-Elemene | C15H24 |
| TCMIP100935 | Hexadecane | C16H34 |
| TCMIP100352 | strumaroside | C35H60O6 |
| TCMIP100310 | nonadecane | C19H40 |
| TCMIP100276 | Hexadecanoic acid | C16H32O2 |
| TCMIP100203 | Stigmasterol | C29H48O |
| TCMIP100176 | β-Caryophyllene | C15H24 |
| TCMIP100168 | ethyl palmitate | C18H36O2 |
| TCMIP100111 | guercetol | C15H10O7 |
| TCMIP100092 | γ-elemene | C15H24 |
| TCMIP100084 | β-sitosterol | C29H50O |
| TCMIP100070 | α-gurjunene | C15H24 |

***Flos Lonicerae* ingredients**

| **compound number** | **name** | **molecular formula** |
| --- | --- | --- |
| TCMIP100054 | (trans-trans)farnesol | C15H26O |
| TCMIP100084 | β-sitosterol | C29H50O |
| TCMIP100140 | carvacrol | C10H14O |
| TCMIP100168 | ethyl palmitate | C18H36O2 |
| TCMIP100185 | Inositol | C6H12O6 |
| TCMIP100186 | Inositol c | C6H12O6 |
| TCMIP100203 | Stigmasterol | C29H48O |
| TCMIP100205 | α-Terpineol | C10H18O |
| TCMIP100231 | 3-caffeoylquinic acid | C16H18O9 |
| TCMIP100352 | strumaroside | C35H60O6 |
| TCMIP100594 | Hederagenin | C30H48O4 |
| TCMIP100853 | α-Pinene | C10H16 |
| TCMIP100874 | luteolin | C15H10O6 |
| TCMIP100879 | phenylethanol | C8H10O |
| TCMIP100883 | geraniol | C10H18O |
| TCMIP101161 | Eugenol | C10H12O2 |
| TCMIP101166 | Linalool | C10H18O |
| TCMIP101508 | Methyl linoleate | C19H34O2 |
| TCMIP103428 | (E)-Aldosecologanin | C34H46O19 |
| TCMIP103429 | (Z)-Aldosecologanin | C34H46O19 |
| TCMIP103430 | 2'-O-[β-D-Apiofuranosyl(1-2)-β-D-glucopyranosyl]isoliquiritigenin | C26H30O14 |
| TCMIP103431 | Benzyl alcohol | C7H8O |
| TCMIP103432 | Benzyl benzoate | C14H12O2 |
| TCMIP103433 | 1,1'-Bicyclohexyl | C12H22 |
| TCMIP103434 | Chrysoeriol | C16H12O6 |
| TCMIP103435 | Citronellol | C10H20O |
| TCMIP103436 | 7-O-(4-β-D-Glucopyranosyloxy-3-methoxybenzoyl) secologanolic acid | C30H40O18 |
| TCMIP103437 | 1-Hexene | C6H12 |
| TCMIP103438 | 6'-O-(7α-Hydroxyswerosyloxy)loganin | C33H46O19 |
| TCMIP103439 | Ioniceroside C | C53H86O22 |
| TCMIP103440 | Isochlorogenic acid | C16H18O9 |
| TCMIP103441 | Linalyl oxide | C10H18O2 |
| TCMIP103442 | Loganic acid | C16H24O10 |
| TCMIP103443 | loganin | C17H26O10 |
| TCMIP103444 | Loniceracetalide A | C21H32O11 |
| TCMIP103445 | Loniceracetalide B | C21H32O11 |
| TCMIP103446 | Lonicerin | C27H30O15 |
| TCMIP103447 | Loniflavone | C30H18O10 |
| TCMIP103448 | Macranthoidin A | C59H96O27 |
| TCMIP103449 | Macranthoidin B | C65H106O32 |
| TCMIP103450 | Macranthoside A | C47H76O17 |
| TCMIP103451 | Macranthoside B | C53H86O22 |
| TCMIP103452 | 2-Methyl-1-butanol | C5H12O |
| TCMIP103453 | 3'-O-Methyl loniflavone | C31H20O10 |
| TCMIP103454 | 3-Methyl-2-(2-pentenyl)-2-cyclopenten-1-one | C11H16O |
| TCMIP103455 | New triterpennoid glycoside | C65H106O32 |
| TCMIP103456 | L-Phenylalaninosecologanin | C26H35NO11 |

***Radix Scutellariae*** ingredients

| **compound number** | **name** | **molecular formula** |
| --- | --- | --- |
| TCMIP100084 | β-sitosterol | C29H50O |
| TCMIP100203 | Stigmasterol | C29H48O |
| TCMIP100276 | Hexadecanoic acid | C16H32O2 |
| TCMIP100350 | sucrose | C12H22O11 |
| TCMIP100617 | Campesterol | C28H48O |
| TCMIP100825 | 4'-Hydroxywogonin | C16H12O6 |
| TCMIP100828 | Scutellarin | C21H18O12 |
| TCMIP100829 | Wogonin | C16H12O5 |
| TCMIP101404 | 5-hydroxy-6,7,8,4'-tetramethoxyflavone | C19H18O8 |
| TCMIP101539 | oroxylin A | C16H12O5 |
| TCMIP101569 | Skullcapflavone I | C17H14O6 |
| TCMIP101888 | Baicalin | C21H18O11 |
| TCMIP102305 | Chrysin | C15H10O4 |
| TCMIP102494 | Isoschaftoside | C26H28O14 |
| TCMIP103213 | 6-C-Arabinopyranosyl-8-C-glucopyranosyl-5,7-dihydroxyflavone | C26H28O13 |
| TCMIP103214 | 8-C-Arabinopyranosyl-6-C-glucopyranosyl-5,7-dihydroxyflavone | C26H28O13 |
| TCMIP103215 | Baicalein | C15H10O5 |
| TCMIP103216 | Baicalein-7-O-β-D-glucopyranoside | C21H20O10 |
| TCMIP103217 | Dihydrobaicalin | C21H20O11 |
| TCMIP103218 | Dihydrooroxylin A | C16H14O5 |
| TCMIP103219 | 5,8-Dihydroxy-6,7-dimethoxyflavone | C17H14O6 |
| TCMIP103220 | 5,7-Dihydroxy-6,8,2',3'-tetramethoxyflavone | C19H18O8 |
| TCMIP103221 | 5,2'-Dihydroxy-6,7,8-trimethoxyflavone | C18H16O7 |
| TCMIP103222 | Eriodictyol | C15H12O6 |
| TCMIP103223 | 5-Hydroxy-7,8-dimethoxyflavone | C17H14O5 |
| TCMIP103224 | 2-(3-Hydroxy-4-methoxyphenyl)-ethyl-1-O-α-L-rhamnosyl-(1-3)-β-D-(4-feruloyl)-glucoside | C31H40O15 |
| TCMIP103225 | Isoscutellarein | C15H10O6 |
| TCMIP103226 | 7-Methoxybaicalein | C16H12O5 |
| TCMIP103227 | 8-Methoxy-5-O-glucoside flavone | C22H22O9 |
| TCMIP103228 | Norwogonin | C15H10O5 |
| TCMIP103229 | Oroxylin A 7-O-glucuronide | C22H20O11 |
| TCMIP103230 | 3,5,7,2'6'-Pentahydroxyflavanone | C15H12O7 |
| TCMIP103231 | 3,5,7,2',6'-Pentahydroxy flavone | C15H10O7 |
| TCMIP103232 | 1-Phenyl-1,3-butanedion | C10H10O2 |
| TCMIP103233 | (E)-4-Phenyl-3-buten-2-one | C10H10O |
| TCMIP103234 | Scutevulin | C16H12O6 |
| TCMIP103235 | 5,7,2',5'-Tetrahydroxy-8,6'-dimethoxy flavone | C17H14O8 |
| TCMIP103236 | 5,7,2',3'-Tetrahydroxyflavone | C15H10O6 |
| TCMIP103237 | 5,7,2',6'-Tetrahydroxyflavone | C15H10O6 |
| TCMIP103238 | 2,6,2',4'-Tetrahydroxy-6’-methoxychalcone | C16H14O6 |
| TCMIP103239 | 5,2',6'-Trihydroxy-7,8-dimethoxyflavone | C17H14O7 |
| TCMIP103240 | 5,7,2'-Trihydroxy-8,6'-dimethoxyflavone | C17H14O7 |
| TCMIP103241 | 5,8,2'-Trihydroxy-6,7-dimethoxyflavone | C17H14O7 |
| TCMIP103242 | 5,7,2'-Trihydroxyflavone | C15H10O5 |
| TCMIP103243 | 5,7,4'-Trihydroxy-6-C-glucoside-8-C-arabinoside flavone | C26H28O14 |
| TCMIP103244 | 7,2',6'-Trihydroxy-5-methoxychalcone | C16H14O5 |
| TCMIP103245 | 5,7,4'-Trihydroxy-6-methoxyflavanone | C16H14O6 |
| TCMIP103246 | 7,2'6'-Trihydroxy-5-methoxyflavanone | C16H14O6 |
| TCMIP103247 | 5,7,2'-Trihydroxy-6-methoxyflavone | C16H12O6 |
| TCMIP103248 | 5,8,2'-Trihydroxy-7-methoxyflavone | C16H12O6 |
| TCMIP103249 | 5,2',5'-Trihydroxy-6,7,8-trimethoxyflavone | C18H16O8 |
| TCMIP103250 | Viscidulin III-6'-O-β-D-glucopyranoside | C23H24O13 |
| TCMIP103251 | Wogonin 5-β-D-glucoside | C22H22O10 |
| TCMIP103252 | Wogonoside | C22H20O11 |

***Radix Paeoniae Rubra*** ingredients

| **compound number** | **name** | **molecular formula** |
| --- | --- | --- |
| TCMIP101417 | (Z)-(IS,5R)-β-Pinen-10-yl-β-vicianoside | C21H34O10 |
| TCMIP101416 | Lactiflorin | C23H26O10 |
| TCMIP101415 | 8-Diebenzoylpaeoniflorin | C16H24O10 |
| TCMIP101414 | benzoyl paeoniflorin | C30H32O12 |
| TCMIP101413 | paeonolide | C20H28O12 |
| TCMIP100551 | 1,2,4,6-Tetra-O-galloyl-β-D-glucose | C34H28O22 |
| TCMIP100542 | Paeonol | C9H10O3 |
| TCMIP100538 | Paeoniflorin | C23H28O11 |
| TCMIP100537 | Paeoniflorigenone | C17H18O6 |
| TCMIP100531 | Oxypaeoniflorin | C23H28O12 |
| TCMIP100525 | Eugeniin | C41H30O26 |
| TCMIP100519 | Albiflorin | C23H28O11 |
| TCMIP100516 | oxypaeoniflorin | C23H28O12 |
| TCMIP100513 | paeonioflorin | C23H28O11 |
| TCMIP100461 | Gallic acid | C7H6O5 |
| TCMIP100352 | strumaroside | C35H60O6 |
| TCMIP100236 | hydroxybenzoic acid | C7H6O3 |
| TCMIP100213 | benzoic acid | C7H6O2 |
| TCMIP100089 | β-amyrin | C30H50O |
| TCMIP100084 | β-sitosterol | C29H50O |

***Fructus Aurantii Immaturus*** ingredients

| **compound number** | **name** | **molecular formula** |
| --- | --- | --- |
| TCMIP106570 | poncirin | C27H32O12 |
| TCMIP106569 | Hesperitin | C16H14O6 |
| TCMIP106568 | 5,7-dihydroxycoumarin | C9H6O4 |
| TCMIP106567 | obaculactone | C26H30O8 |
| TCMIP106566 | obacunone | C26H30O7 |
| TCMIP106565 | Naringin | C27H32O14 |
| TCMIP106564 | naringenin-7-rutinoside | C27H32O14 |
| TCMIP106563 | naringenin-4'-glucoside-7-rutinoside | C33H42O19 |
| TCMIP106562 | limonin | C25H28O8 |
| TCMIP106561 | isosakuranetin-7-rutinoside | C28H34O14 |
| TCMIP106560 | 6-methoxyaurapten | C20H24O4 |
| TCMIP106559 | ichangin | C27H34O8 |
| TCMIP106558 | eriocitrin | C27H32O15 |
| TCMIP106557 | citrusin B | C27H36O13 |
| TCMIP106556 | aurapten | C19H22O3 |
| TCMIP106555 | 7-geranyloxycoumarin | C19H22O3 |
| TCMIP106340 | 5,7,4'-Trimethoxyflavone | C18H16O5 |
| TCMIP106339 | 5,7,8,4'-Tetramethoxyflavone | C19H18O6 |
| TCMIP106338 | Nomilinic acid glucoside | C34H48O16 |
| TCMIP106337 | Nomilinic acid | C28H36O10 |
| TCMIP106336 | Neohesperidin | C28H34O15 |
| TCMIP106335 | Isolimonic acid (16-17)lactone | C26H32O9 |
| TCMIP106334 | 19-Hydroxydeacetylnomilinic acid-17-β-D-glucopyranoside | C32H46O16 |
| TCMIP106330 | N-Methyltyramine | C9H13NO |
| TCMIP105525 | Synephrine | C9H13NO2 |
| TCMIP104465 | Isosinensetin | C20H20O7 |
| TCMIP103607 | Nomilin | C28H34O9 |
| TCMIP103539 | Sinensetin | C20H20O7 |
| TCMIP103446 | Lonicerin | C27H30O15 |
| TCMIP103027 | Naringenin | C15H12O5 |
| TCMIP102744 | Naringin | C27H32O14 |
| TCMIP102211 | Apigenin-7-O-neohesperidoside | C27H30O14 |
| TCMIP102188 | Isopimpinellin | C13H10O5 |
| TCMIP101587 | coniferin | C16H22O8 |
| TCMIP101408 | hesperidin | C28H34O15 |
| TCMIP101392 | terpinolene | C10H16 |
| TCMIP101387 | 5,7,8,3',4'-pentamethoxyflavone | C20H20O7 |
| TCMIP101386 | nobiletin | C21H22O8 |
| TCMIP101126 | p-cymol | C10H14 |
| TCMIP101079 | Limonin | C26H30O8 |
| TCMIP100666 | Imperatorin | C16H14O4 |
| TCMIP100646 | bergapten | C12H8O4 |
| TCMIP100228 | linalool | C10H18O |
| TCMIP100205 | α-Terpineol | C10H18O |
| TCMIP100170 | 4-terpineol | C10H18O |
| TCMIP100139 | sabinene | C10H16 |
| TCMIP100128 | limonene | C10H16 |
| TCMIP100102 | scopoletin | C10H8O4 |
| TCMIP100024 | α-pinene | C10H16 |

***Cortex Mori* ingredients**

| **compound number** | **name** | **molecular formula** |
| --- | --- | --- |
| TCMIP107019 | kuwanon Z | C34H26O10 |
| TCMIP107018 | kuwanon Y | C34H30O9 |
| TCMIP107017 | kuwanon L | C35H30O11 |
| TCMIP107016 | kuwanon K | C40H36O11 |
| TCMIP107015 | kuwanon F | C25H26O6 |
| TCMIP107014 | kuwanon E | C25H28O6 |
| TCMIP107013 | kuwanon D | C25H26O6 |
| TCMIP107012 | kuwanon B | C25H24O6 |
| TCMIP107011 | sanggenone P | C45H44O12 |
| TCMIP107010 | sanggenone O | C40H36O12 |
| TCMIP107009 | sanggenone N | C25H26O6 |
| TCMIP107008 | sanggenone M | C25H24O7 |
| TCMIP107007 | sanggenone L | C30H32O7 |
| TCMIP107006 | sanggenone K | C30H32O6 |
| TCMIP107005 | sanggenone J | C30H32O6 |
| TCMIP107004 | sanggenone I | C25H26O6 |
| TCMIP107003 | sanggenone H | C20H18O6 |
| TCMIP107002 | sanggenone G | C40H38O11 |
| TCMIP107001 | sanggenone F | C21H20O5 |
| TCMIP107000 | sanggenone E | C45H44O12 |
| TCMIP106999 | sanggenone D | C40H36O12 |
| TCMIP106998 | sanggenone C | C40H36O12 |
| TCMIP106997 | sanggenone B | C33H30O9 |
| TCMIP106996 | mulberrofuran K | C34H26O8 |
| TCMIP106995 | mulberrofuran B | C25H28O4 |
| TCMIP106994 | kuwanon H / moracenin A / albanin G | C45H44O11 |
| TCMIP106993 | kuwanon A | C25H24O6 |
| TCMIP106992 | kuwanon I | C40H38O10 |
| TCMIP106991 | sanggenone A | C25H24O7 |
| TCMIP106990 | moracenin C | C45H44O11 |
| TCMIP106989 | oxydihydromorusin | C25H26O7 |
| TCMIP106988 | cyclomulberrin | C25H24O6 |
| TCMIP106987 | Multiflor-7-ene | C30H50 |
| TCMIP106986 | Mulberrofuran Q | C34H24O10 |
| TCMIP106985 | Mulberrofuran P | C34H22O9 |
| TCMIP106984 | Mulberrofuran O | C39H34O9 |
| TCMIP106983 | Mulberrofuran N | C25H28O4 |
| TCMIP106982 | Mulberrofuran M | C34H22O10 |
| TCMIP106981 | Mulberrofuran C | C34H28O9 |
| TCMIP106980 | Moscatin | C15H12O3 |
| TCMIP106979 | Moracenin D | C40H38O12 |
| TCMIP106978 | Cyclomulberrochromene | C25H22O6 |
| TCMIP106977 | 1'-Methoxy-2'-hydroxydihydromollugin | C18H20O6 |
| TCMIP106543 | / kuwanon I | C40H38O10 |
| TCMIP104994 | 4'-Prenyloxyresveratrol | C19H20O4 |
| TCMIP104983 | (6S,9R)-Roseoside | C19H30O8 |
| TCMIP104982 | Pentanic acid | C5H10O2 |
| TCMIP104974 | Moracin D | C19H16O4 |
| TCMIP102222 | (+)-Cycloolivil | C20H24O7 |
| TCMIP101739 | quercetin | C15H10O7 |
| TCMIP101021 | D-Borneol | C10H18O |
| TCMIP100506 | Sanguinarine | C20H14NO4 |
| TCMIP100102 | scopoletin | C10H8O4 |
| TCMIP100051 | Umbelliferone | C9H6O3 |

***Semen Descurainiae* ingredients**

| **compound number** | **name** | **molecular formula** |
| --- | --- | --- |
| TCMIP105375 | trans-Sinapic acid glucoside | C17H22O10 |
| TCMIP105374 | cis-Sinapic acid glucoside | C17H22O10 |
| TCMIP105373 | Sinapic acid ethyl ester | C13H16O5 |
| TCMIP105372 | Quercetin-7-O-β-D-glucopyranosyl(1→6)-β-D-glucopyranoside | C27H30O17 |
| TCMIP105371 | 4-Pentenamide | C5H9NO |
| TCMIP105370 | Kaempferol-3-β-D-glucopyranosyl-7-O-[(2-O-trans-sinnapoyl)-β-D-glucopyranosyl (1→6)]-β-D-glucopyranoside | C44H50O25 |
| TCMIP105369 | Isorhamnetin-3-O-glucoside | C22H22O12 |
| TCMIP105368 | Isorhamnetin-7-β-D-gentiobioside | C28H32O17 |
| TCMIP105367 | Helveticoside | C29H42O9 |
| TCMIP105366 | Descurainolide B | C21H22O8 |
| TCMIP105365 | Descurainolide A | C13H16O5 |
| TCMIP105364 | Descurainin | C16H18O6 |
| TCMIP104038 | Strophanthidin | C23H32O6 |
| TCMIP103816 | Erucic acid | C22H42O2 |
| TCMIP103375 | Benzyl isothiocyanate | C8H7NS |
| TCMIP103374 | Allyl isothiocyanate | C4H5NS |
| TCMIP101758 | Allyl disulfide | C6H10S2 |
| TCMIP100775 | Sinalbine | C14H19NO10S2 |
| TCMIP100406 | Isoquercitrin | C21H20O12 |
| TCMIP100385 | Isorhamnetin | C16H12O7 |
| TCMIP100171 | 7-methoxycoumarin | C10H8O3 |

**Supplementary Figure 2**

**Potential target information of Yiqi Huayu Jiedu decoction**

| ID | gene name | protein name |
| --- | --- | --- |
| P40939 | HADHA | Trifunctional enzyme subunit alpha, mitochondrial |
| Q16836 | HADH | Hydroxyacyl-coenzyme A dehydrogenase, mitochondrial |
| Q05655 | PRKCD | Protein kinase C delta type |
| O60513 | B4GALT4 | Beta-1,4-galactosyltransferase 4 |
| P04637 | TP53 | Cellular tumor antigen p53 |
| O60909 | B4GALT2 | Beta-1,4-galactosyltransferase 2 |
| P07327 | ADH1A | Alcohol dehydrogenase |
| P30084 | ECHS1 | Enoyl-CoA hydratase, mitochondrial |
| P07900 | HSP90AA1 | Heat shock protein HSP 90-alpha |
| Q08462 | ADCY2 | Adenylate cyclase type 2 |
| P12931 | SRC | Proto-oncogene tyrosine-protein kinase Src |
| Q9UKG9 | CROT | Peroxisomal carnitine O-octanoyltransferase |
| P78368 | CSNK1G2 | Casein kinase I isoform gamma-2 |
| P54646 | PRKAA2 | 5'-AMP-activated protein kinase catalytic subunit alpha-2 |
| P00326 | ADH1C | Alcohol dehydrogenase 1C |
| P62873 | GNB1 | Guanine nucleotide-binding protein G(I)/G(S)/G(T) subunit beta-1 |
| P05023 | ATP1A1 | Sodium/potassium-transporting ATPase subunit alpha-1 |
| Q13131 | PRKAA1 | AMPK subunit alpha-1 |
| O95622 | ADCY5 | Adenylate cyclase type 5 |
| P05412 | JUN | Transcription factor AP-1 |
| P06241 | FYN | Tyrosine-protein kinase Fyn |
| P63000 | RAC1 | Ras-related C3 botulinum toxin substrate 1 |
| P30872 | SSTR1 | Somatostatin receptor type 1 |
| Q9NR33 | POLE4 | DNA polymerase epsilon subunit 4 |
| P49902 | NT5C2 | Cytosolic purine 5'-nucleotidase |
| P19838 | NFKB1 | Nuclear factor NF-kappa-B p105 subunit |
| P49419 | ALDH7A1 | Alpha-aminoadipic semialdehyde dehydrogenase |
| Q04206 | RELA | Transcription factor p65 |
| Q08188 | TGM3 | Protein-glutamine gamma-glutamyltransferase E |
| P00519 | ABL1 | Tyrosine-protein kinase ABL1 |
| Q00653 | NFKB2 | Nuclear factor NF-kappa-B p100 subunit |
| P63096 | GNAI1 | Guanine nucleotide-binding protein G(i) subunit alpha-1 |
| P15954 | COX7C | Cytochrome c oxidase subunit 7C |
| P11142 | HSPA8 | Heat shock cognate 71 kDa protein |
| Q13363 | CTBP1 | C-terminal-binding protein 1 |
| P30411 | BDKRB2 | B2 bradykinin receptor |
| P17612 | PRKACA | cAMP-dependent protein kinase catalytic subunit alpha |
| P61586 | RHOA | Transforming protein RhoA |
| P49915 | GMPS | GMP synthase [glutamine-hydrolyzing] |
| P68104 | EEF1A1 | Elongation factor 1-alpha 1 |
| O14920 | IKBKB | Inhibitor of nuclear factor kappa-B kinase subunit beta |
| P13501 | CCL5 | C-C motif chemokine 5 |
| Q9Y478 | PRKAB1 | 5'-AMP-activated protein kinase subunit beta-1 |
| P32745 | SSTR3 | Somatostatin receptor type 3 |
| P08588 | ADRB1 | Beta-1 adrenergic receptor |
| P0DMS8 | ADORA3 | Adenosine receptor A3 |
| Q9NRF9 | POLE3 | DNA polymerase epsilon subunit 3 |
| P25963 | NFKBIA | NF-kappa-B inhibitor alpha |
| P19367 | HK1 | Hexokinase-1 |
| Q9BTZ2 | DHRS4 | Dehydrogenase/reductase SDR family member 4 |
| P10415 | BCL2 | Apoptosis regulator Bcl-2 |
| P11226 | MBL2 | Mannose-binding protein C |
| P09884 | POLA1 | DNA polymerase alpha catalytic subunit |
| P35346 | SSTR5 | Somatostatin receptor type 5 |
| P50416 | CPT1A | Carnitine O-palmitoyltransferase 1 |
| P18464 | HLA-B | HLA class I histocompatibility antigen |
| P61073 | CXCR4 | C-X-C chemokine receptor type 4 |
| P41146 | OPRL1 | Nociceptin receptor |
| P21554 | CNR1 | Cannabinoid receptor 1 |
| Q9BY32 | ITPA | Inosine triphosphate pyrophosphatase |
| P25024 | CXCR1 | C-X-C chemokine receptor type 1 |
| P22392 | NME2 | Nucleoside diphosphate kinase B |
| P06730 | EIF4E | Eukaryotic translation initiation factor 4E |
| P06239 | LCK | Tyrosine-protein kinase Lck |
| Q15738 | NSDHL | terol-4-alpha-carboxylate 3-dehydrogenase, decarboxylating |
| O14936 | CASK | Peripheral plasma membrane protein CASK |
| Q13627 | DYRK1A | Dual specificity tyrosine-phosphorylation-regulated kinase 1A |
| P35557 | GCK | Glucokinase |
| P51648 | ALDH3A2 | Fatty aldehyde dehydrogenase |
| P27986 | PIK3R1 | Phosphatidylinositol 3-kinase regulatory subunit alpha |
| P36542 | ATP5C1 | ATP synthase subunit gamma, mitochondrial |
| P68366 | TUBA4A | Tubulin alpha-4A chain |
| P35570 | IRS1 | Insulin receptor substrate 1 |
| P51003 | PAPOLA | Poly(A) polymerase alpha |
| P62330 | ARF6 | ADP-ribosylation factor 6 |
| P41091 | EIF2S3 | Eukaryotic translation initiation factor 2 subunit 3 |
| P13639 | EEF2 | Elongation factor 2 |
| P11310 | ACADM | Medium-chain specific acyl-CoA dehydrogenase |
| Q07864 | POLE | DNA polymerase epsilon catalytic subunit A |
| P62714 | PPP2CB | Serine/threonine-protein phosphatase 2A catalytic subunit beta isoform |
| P10275 | AR | Androgen receptor |
| P06881 | CALCA | Calcitonin gene-related peptide 1 |
| ACAD8 | ACAD8 | Isobutyryl-CoA dehydrogenase, mitochondrial |
| P68400 | CSNK2A1 | Casein kinase II subunit alpha |
| Q08493 | PDE4C | cAMP-specific 3',5'-cyclic phosphodiesterase 4C |
| P60953 | CDC42 | Cell division control protein 42 homolog |
| P56282 | POLE2 | DNA polymerase epsilon subunit 2 |
| Q9Y286 | SIGLEC7 | Sialic acid-binding Ig-like lectin 7 |
| Q06609 | RAD51 | DNA repair protein RAD51 homolog 1 |
| P49247 | RPIA | Ribose-5-phosphate isomerase |
| P04150 | NR3C1 | Glucocorticoid receptor |
| P15531 | NME1 | Nucleoside diphosphate kinase A |
| Q9BXI2 | SLC25A2 | Mitochondrial ornithine transporter 2 |
| P24310 | COX7A1 | ytochrome c oxidase subunit 7A1, mitochondrial |
| P25098 | ADRBK1 | Beta-adrenergic receptor kinase 1 |
| P43119 | PTGIR | Prostacyclin receptor |
| O14561 | NDUFAB1 | Acyl carrier protein, mitochondrial |
| P06576 | ATP5B | ATP synthase subunit beta, mitochondrial |
| P04053 | DNTT | DNA nucleotidylexotransferase |
| P00568 | AK1 | Adenylate kinase isoenzyme 1 |
| P30518 | AVPR2 | Vasopressin V2 receptor |
| P49368 | CCT3 | T-complex protein 1 subunit gamma |
| P11388 | TOP2A | DNA topoisomerase 2-alpha |
| P04406 | GAPDH | Glyceraldehyde-3-phosphate dehydrogenase |
| P05771 | PRKCB | Protein kinase C beta type |
| P00491 | PNP | Purine nucleoside phosphorylase |
| P40616 | ARL1 | ADP-ribosylation factor-like protein 1 |
| O95865 | DDAH2 | N(G),N(G)-dimethylarginine dimethylaminohydrolase 2 |
| P13945 | ADRB3 | Beta-3 adrenergic receptor |
| P47985 | UQCRFS1 | Cytochrome b-c1 complex subunit Rieske, mitochondrial |
| P36871 | PGM1 | Phosphoglucomutase-1, PGM 1 |
| P54819 | AK2 | Adenylate kinase 2, mitochondrial |
| Q9Y233 | PDE10A | cAMP and cAMP-inhibited cGMP 3',5'-cyclic phosphodiesterase 10A |
| P16581 | SELE | E-selectin |
| P22888 | LHCGR | Lutropin-choriogonadotropic hormone receptor |
| P18509 | ADCYAP1 | Pituitary adenylate cyclase-activating polypeptide |
| P08473 | MME | Neprilysin |
| P25786 | PSMA1 | Proteasome subunit alpha type-1 |
| P60900 | PSMA6 | Proteasome subunit alpha type-6 |
| P14621 | ACYP2 | Acylphosphatase-2 |
| Q92769 | HDAC2 | Histone deacetylase 2 |
| P11233 | RALA | Ras-related protein Ral-A |
| P47712 | PLA2G4A | Cytosolic phospholipase A2 |
| P49720 | PSMB3 | Proteasome subunit beta type-3 |
| P62826 | RAN | GTP-binding nuclear protein Ran |
| P19404 | NDUFV2 | NADH dehydrogenase [ubiquinone] flavoprotein 2, mitochondrial |
| Q16795 | NDUFA9 | NADH dehydrogenase [ubiquinone] 1 alpha subcomplex subunit 9, mitochondrial |
| P20674 | COX5A | Cytochrome c oxidase subunit 5A, mitochondrial |
| P03901 | MT-ND4L | NADH-ubiquinone oxidoreductase chain 4L |
| P43088 | PTGFR | Prostaglandin F2-alpha receptor |
| P05091 | ALDH2 | Aldehyde dehydrogenase, mitochondrial |
| P63208 | SKP1 | S-phase kinase-associated protein 1 |
| P40926 | MDH2 | Malate dehydrogenase, mitochondrial |
| P36873 | PPP1CC | Serine/threonine-protein phosphatase PP1-gamma catalytic subunit |
| P09874 | PARP1 | Poly [ADP-ribose] polymerase 1 |
| Q9Y5K3 | PCYT1B | Choline-phosphate cytidylyltransferase B |
| P25788 | PSMA3 | Proteasome subunit alpha type-3 |
| P42684 | ABL2 | Tyrosine-protein kinase ABL2 |
| P03915 | MT-ND5 | NADH-ubiquinone oxidoreductase chain 5 |
| O75489 | NDUFS3 | NADH dehydrogenase [ubiquinone] iron-sulfur protein 3, mitochondrial |
| O76083 | PDE9A | High affinity cGMP-specific 3',5'-cyclic phosphodiesterase 9A |
| Q13423 | NNT | NAD(P) transhydrogenase, mitochondrial |
| O75380 | NDUFS6 | NADH dehydrogenase [ubiquinone] iron-sulfur protein 6, mitochondrial |
| P49821 | NDUFV1 | NADH dehydrogenase [ubiquinone] flavoprotein 1,mitochondrial |
| O95182 | NDUFA7 | NADH dehydrogenase [ubiquinone] 1 alpha subcomplex subunit 7 |
| P35243 | RCVRN | Recoverin |
| P20618 | PSMB1 | Proteasome subunit beta type-1 |
| P07947 | YES1 | Tyrosine-protein kinase Yes |
| Q9Y2Q3 | GSTK1 | Glutathione S-transferase kappa 1 |
| P23921 | RRM1 | Ribonucleoside-diphosphate reductase large subunit |
| P09211 | GSTP1 | Glutathione S-transferase P |
| P30038 | ALDH4A1 | Delta-1-pyrroline-5-carboxylate dehydrogenase, mitochondrial |
| O14939 | PLD2 | Phospholipase D2, PLD 2 |
| O94788 | ALDH1A2 | Retinal dehydrogenase 2 |
| Q15274 | QPRT | Nicotinate-nucleotide pyrophosphorylase |
| P00338 | LDHA | L-lactate dehydrogenase A chain |
| P28072 | PSMB6 | Proteasome subunit beta type-6 |
| Q16665 | HIF1A | Hypoxia-inducible factor 1-alpha |
| P28070 | PSMB4 | Proteasome subunit beta type-4 |
| P31350 | RRM2 | Ribonucleoside-diphosphate reductase subunit M2 |
| P12268 | IMPDH2 | Inosine-5'-monophosphate dehydrogenase 2 |
| P07195 | LDHB | L-lactate dehydrogenase B chain |
| P10636 | MAPT | Microtubule-associated protein tau |
| P07237 | P4HB | Protein disulfide-isomerase |
| Q13224 | GRIN2B | Glutamate receptor ionotropic |
| O60488 | ACSL4 | Long-chain-fatty-acid--CoA ligase 4 |
| ACOX1 | ACOX1 | Peroxisomal acyl-coenzyme A oxidase 1 |
| P55072 | VCP | Transitional endoplasmic reticulum ATPase |
| Q09428 | ABCC8 | ATP-binding cassette sub-family C member 8 |
| O14788 | TNFSF11 | Tumor necrosis factor ligand superfamily member 11 |
| Q03393 | PTS | 6-pyruvoyl tetrahydrobiopterin synthase |
| P14854 | COX6B1 | Cytochrome c oxidase subunit 6B1 |
| P19793 | RXRA | Retinoic acid receptor RXR-alpha |
| Q16774 | GUK1 | Guanylate kinase |
| P28074 | PSMB5 | Proteasome subunit beta type-5 |
| P16455 | MGMT | Methylated-DNA--protein-cysteine methyltransferase |
| P07814 | EPRS | Bifunctional glutamate/proline--tRNA ligase |
| P27708 | CAD | CAD protein |
| P09669 | COX6C | Cytochrome c oxidase subunit 6C |
| P08581 | HGF | Hepatocyte growth factor receptor |
| P06746 | POLB | DNA polymerase beta |
| P49189 | ALDH9A1 | 4-trimethylaminobutyraldehyde dehydrogenase |
| O14818 | PSMA7 | Proteasome subunit alpha type-7 |
| P48507 | GCLM | Glutamate--cysteine ligase regulatory subunit |
| P11387 | TOP1 | DNA topoisomerase 1 |
| Q13200 | PSMD2 | 26S proteasome non-ATPase regulatory subunit 2 |
| P49721 | PSMB2 | Proteasome subunit beta type-2 |
| P51692 | STAT5B | Signal transducer and activator of transcription 5B |
| Q99460 | PSMD1 | 26S proteasome non-ATPase regulatory subunit 1 |
| Q9UKV0 | HDAC9 | Histone deacetylase 9 |
| P00352 | ALDH1A1 | Retinal dehydrogenase 1 |
| P31939 | ATIC | Bifunctional purine biosynthesis protein PURH |
| Q99873 | PRMT1 | Protein arginine N-methyltransferase 1 |
| P10276 | RARA | Retinoic acid receptor alpha |
| Q02218 | OGDH | 2-oxoglutarate dehydrogenase, mitochondrial |
| P67775 | PPP2CA | Serine/threonine-protein phosphatase 2A catalytic subunit alpha isoform |
| O75390 | CS | Citrate synthase, mitochondrial |
| Q05329 | GAD2 | Glutamate decarboxylase 2 |
| P30837 | ALDH1B1 | Aldehyde dehydrogenase X, mitochondrial |
| P31749 | AKT1 | RAC-alpha serine/threonine-protein kinase |
| P15291 | B4GALT1 | Beta-1,4-galactosyltransferase 1 |
| Q99714 | HSD17B10 | 3-hydroxyacyl-CoA dehydrogenase type-2 |
| Q08426 | EHHADH | Peroxisomal bifunctional enzyme |
| O15111 | CHUK | Inhibitor of nuclear factor kappa-B kinase subunit alpha, I-kappa-B kinase alpha |
| P48552 | NRIP1 | Nuclear receptor-interacting protein 1 |
| P15121 | AKR1B1 | Aldose reductase |
| P05113 | IL5 | Interleukin-5 |
| P25789 | PSMA4 | Proteasome subunit alpha type-4 |
| P21589 | NT5E | 5'-nucleotidase |
| P21980 | TGM2 | Protein-glutamine gamma-glutamyltransferase 2 |
| Q99661 | KIF2C | Kinesin-like protein KIF2C |
| P10768 | ESD | S-formylglutathione hydrolase |
| P00390 | GSR | Glutathione reductase, mitochondrial |
| P10826 | RARB | Retinoic acid receptor beta |
| O00141 | SGK1 | Serine/threonine-protein kinase Sgk1 |
| P01574 | IFNB1 | Interferon beta |
| O15217 | GSTA4 | Glutathione S-transferase A4 |
| O14727 | APAF1 | Apoptotic protease-activating factor 1 |
| P63092 | GNAS | Guanine nucleotide-binding protein G(s) subunit alpha isoforms short |
| P09622 | DLD | Dihydrolipoyl dehydrogenase, mitochondrial |
| Q92947 | GCDH | Glutaryl-CoA dehydrogenase, mitochondrial |
| P22695 | UQCRC2 | Cytochrome b-c1 complex subunit 2, mitochondrial |
| P23786 | CPT2 | Carnitine O-palmitoyltransferase 2, mitochondrial |
| P31150 | GDI1 | Rab GDP dissociation inhibitor alpha |
| P20823 | HNF1A | Hepatocyte nuclear factor 1-alpha |
| P37231 | PPARG | Peroxisome proliferator-activated receptor gamma |
| O60706 | ABCC9 | ATP-binding cassette sub-family C member 9 |
| P51812 | RPS6KA3 | Ribosomal protein S6 kinase alpha-3 |
| P08559 | PDHA1 | Pyruvate dehydrogenase E1 component subunit alpha, somatic form, mitochondrial |
| P30273 | FCER1G | High affinity immunoglobulin epsilon receptor subunit gamma |
| P30520 | ADSS | Adenylosuccinate synthetase isozyme 2 |
| P02768 | ALB | Serum albumin |
| P42262 | GRIA2 | Glutamate receptor 2 |
| P48058 | GRIA4 | Glutamate receptor 4 |
| O14735 | CDIPT | CDP-diacylglycerol--inositol 3-phosphatidyltransferase |
| Q15648 | MED1 | Mediator of RNA polymerase II transcription subunit 1 |
| Q15382 | RHEB | GTP-binding protein Rheb |
| Q01581 | HMGCS1 | Hydroxymethylglutaryl-CoA synthase |
| P21266 | GSTM3 | Glutathione S-transferase Mu 3 |
| P06396 | GSN | Gelsolin |
| P11473 | VDR | Vitamin D3 receptor |
| P08620 | FGF4 | Fibroblast growth factor 4 |
| P08700 | IL3 | Interleukin-3 |
| P22234 | PAICS | Multifunctional protein ADE2 |
| Q02880 | TOP2B | DNA topoisomerase 2-beta |
| P42261 | GRIA1 | Glutamate receptor 1 |
| P29803 | PDHA2 | Pyruvate dehydrogenase E1 component subunit alpha, testis-specific form, mitochondrial |
| P29474 | NOS3 | Nitric oxide synthase, endothelial |
| P46459 | NSF | Vesicle-fusing ATPase |
| P05230 | FGF1 | Fibroblast growth factor 1 |
| P27816 | MAP4 | Microtubule-associated protein 4 |
| P25705 | ATP5A1 | ATP synthase subunit alpha, mitochondrial |
| P23258 | TUBG1 | Tubulin gamma-1 chain |
| O15067 | PFAS | Phosphoribosylformylglycinamidine synthase |
| P19320 | VCAM1 | Vascular cell adhesion protein 1 |
| P07384 | CAPN1 | Calpain-1 catalytic subunit |
| O75746 | SLC25A12 | Calcium-binding mitochondrial carrier protein Aralar1 |
| P14625 | HSP90B1 | Endoplasmin |
| P35354 | PTGS2 | Prostaglandin G/H synthase 2 |
| P00367 | GLUD1 | Glutamate dehydrogenase 1, mitochondrial |
| P10398 | ARAF | Serine/threonine-protein kinase A-Raf |
| P43004 | SLC1A2 | Excitatory amino acid transporter 2 |
| P28482 | MAPK1 | Mitogen-activated protein kinase 1 |
| P62258 | YWHAE | 14-3-3 protein epsilon |
| Q06124 | PTPN11 | Tyrosine-protein phosphatase non-receptor type 11 |
| P10253 | GAA | Lysosomal alpha-glucosidase |
| P01116 | KRAS | GTPase Kras |
| O00459 | PIK3R2 | Phosphatidylinositol 3-kinase regulatory subunit beta |
| P01308 | INS | Insulin |
| O14964 | HGS | Hepatocyte growth factor-regulated tyrosine kinase substrate |
| P62491 | RAB11A | Ras-related protein Rab-11A |
| P51606 | RENBP | N-acylglucosamine 2-epimerase, AGE |
| Q92993 | KAT5 | Histone acetyltransferase KAT5 |
| O00555 | CACNA1A | Voltage-dependent P/Q-type calcium channel subunit alpha-1A |
| P31153 | MAT2A | S-adenosylmethionine synthase isoform type-2 |
| P62942 | FKBP1A | Peptidyl-prolyl cis-trans isomerase FKBP1A |
| O43708 | GSTZ1 | Maleylacetoacetate isomerase |
| P15259 | PGAM2 | Phosphoglycerate mutase 2 |

**Supplementary Figure 3**

**GO enrichment analysis of drug prediction target**

| **ID** | **Term** | **counts** | **P-Value** |
| --- | --- | --- | --- |
| GO:0055114 | oxidation-reduction process | 161 | 1.48×10^-72^ |
| GO:0006120 | mitochondrial electron transport | 45 | 6.71×10^-54^ |
| GO:0032981 | mitochondrial respiratory chain complex I assembly | 42 | 3.74×10^-40^ |
| GO:0006749 | glutathione metabolic process | 26 | 5.81×10^-20^ |
| GO:0086010 | membrane depolarization during action potential | 19 | 1.23×10^-18^ |
| GO:0098869 | cellular oxidant detoxification | 25 | 2.64×10^-16^ |
| GO:0004029 | aldehyde dehydrogenase (NAD) activity | 14 | 2.09×10^-15^ |
| GO:0005248 | voltage-gated sodium channel activity | 14 | 7.58×10^-15^ |
| GO:0005524 | ATP binding | 146 | 8.54×10^-15^ |
| GO:1902600 | hydrogen ion transmembrane transport | 24 | 1.54×10^-14^ |
| GO:0002223 | stimulatory C-type lectin receptor signaling pathway | 29 | 2.48×10^-14^ |
| GO:0070588 | calcium ion transmembrane transport | 30 | 2.21×10^-13^ |
| GO:0006521 | regulation of cellular amino acid metabolic process | 20 | 2.36×10^-13^ |
| GO:0004298 | threonine-type endopeptidase activity | 14 | 4.39×10^-13^ |
| GO:0006979 | response to oxidative stress | 27 | 5.89×10^-13^ |
| GO:0019899 | enzyme binding | 49 | 3.28×10^-12^ |
| GO:0008202 | steroid metabolic process | 16 | 5.89×10^-12^ |
| GO:0038061 | NIK/NF-kappaB signaling | 21 | 6.85×10^-12^ |
| GO:0007215 | glutamate receptor signaling pathway | 11 | 7.13×10^-12^ |
| GO:0005245 | voltage-gated calcium channel activity | 16 | 4.17×10^-11^ |
| GO:0003954 | NADH dehydrogenase activity | 8 | 4.77×10^-11^ |
| GO:0019233 | sensory perception of pain | 19 | 5.43×10^-11^ |
| GO:0050852 | T cell receptor signaling pathway | 30 | 1.43×10^-10^ |
| GO:0006090 | pyruvate metabolic process | 11 | 3.26×10^-10^ |
| GO:0032496 | response to lipopolysaccharide | 32 | 3.85×10^-10^ |
| GO:0003924 | GTPase activity | 37 | 5.53×10^-10^ |
| GO:0001666 | response to hypoxia | 31 | 9.05×10^-10^ |
| GO:0004679 | AMP-activated protein kinase activity | 7 | 9.34×10^-10^ |
| GO:0006809 | nitric oxide biosynthetic process | 9 | 1.44×10^-9^ |
| GO:0019228 | neuronal action potential | 12 | 4.54×10^-9^ |

**Supplementary figure 4**

**KEGG** enrichment analysis of drug prediction target

| **ID** | **Term** | **counts** | **P-Value** |
| --- | --- | --- | --- |
| hsa00190 | Oxidative phosphorylation | 60 | 2.56×10^-23^ |
| hsa00052 | Galactose metabolism | 16 | 3.58×10^-8^ |
| hsa00520 | Amino sugar and nucleotide sugar metabolism | 20 | 4.56×10^-8^ |
| hsa04066 | HIF-1 signaling pathway | 28 | 1.11×10^-5^ |
| hsa04150 | mTOR signaling pathway | 17 | 2.37×10^-4^ |
| hsa00020 | Citrate cycle (TCA cycle) | 11 | 2.58×10^-4^ |
| hsa04080 | Neuroactive ligand-receptor interaction | 50 | 3.86×10^-4^ |
| hsa04210 | Apoptosis | 21 | 4.47×10^-4^ |
| hsa03320 | PPAR signaling pathway | 18 | 4.80×10^-4^ |
| hsa04728 | Dopaminergic synapse | 28 | 5.88×10^-4^ |
| hsa01212 | Fatty acid metabolism | 14 | 6.01×10^-4^ |
| hsa04960 | Aldosterone-regulated sodium reabsorption | 12 | 8.64×10^-4^ |
| hsa05222 | Small cell lung cancer | 20 | 1.17×10^-3^ |
| hsa05221 | Acute myeloid leukemia | 15 | 1.26×10^-3^ |
| hsa04370 | VEGF signaling pathway | 15 | 2.63×10^-3^ |
| hsa04540 | Gap junction | 19 | 4.34×10^-3^ |
| hsa04152 | AMPK signaling pathway | 24 | 5.54×10^-3^ |
| hsa04662 | B cell receptor signaling pathway | 16 | 5.67×10^-3^ |
| hsa00240 | Pyrimidine metabolism | 21 | 6.22×10^-3^ |
| hsa04022 | cGMP - PKG signaling pathway | 30 | 6.48×10^-3^ |
| hsa04919 | Thyroid hormone signaling pathway | 23 | 6.66×10^-3^ |
| hsa04750 | Inflammatory mediator regulation of TRP channels | 20 | 6.67×10^-3^ |
| hsa04064 | NF-kappa B signaling pathway | 18 | 1.21×10^-2^ |
| hsa04068 | FoxO signaling pathway | 24 | 1.32×10^-2^ |
| hsa04014 | Ras signaling pathway | 37 | 1.33×10^-2^ |
| hsa04010 | MAPK signaling pathway | 41 | 1.35×10^-2^ |
| hsa04660 | T cell receptor signaling pathway | 19 | 2.28×10^-2^ |
| hsa04664 | Fc epsilon RI signaling pathway | 14 | 2.30×10^-2^ |
| hsa04270 | Vascular smooth muscle contraction | 21 | 2.93×10^-2^ |
| hsa00970 | Aminoacyl-tRNA biosynthesis | 13 | 3.13×10^-2^ |

**Supplementary figure 5**

**The target information for ARDS diseases**

| ID | gene name | protein name |
| --- | --- | --- |
| Q02750 | MAP2K1 | Dual specificity mitogen-activated protein kinase kinase 1 |
| Q9NQ66 | PLCB1 | 1-phosphatidylinositol 4,5-bisphosphate phosphodiesterase beta-1 |
| Q92574 | TSC1 | Hamartin |
| Q06787 | FMR1 | Synaptic functional regulator FMR1 |
| P42336 | PIK3CA | Phosphatidylinositol 4,5-bisphosphate 3-kinase catalytic subunit alpha isoform |
| P04049 | RAF1 | RAF proto-oncogene serine/threonine-protein kinase |
| Q9Y243 | AKT3 | RAC-gamma serine/threonine-protein kinase |
| P11274 | BCR | Breakpoint cluster region protein |
| P01112 | HRAS | GTPase Hras |
| P78347 | GTF2I | General transcription factor II-I |
| Q92793 | CREBBP | CREB-binding protein |
| Q9NQ11 | ATP13A2 | Cation-transporting ATPase 13A2 |
| P01189 | POMC | Pro-opiomelanocortin |
| P12004 | PCNA | Proliferating cell nuclear antigen |
| P06400 | RB1 | Retinoblastoma-associated protein |
| P43034 | PAFAH1B1 | Platelet-activating factor acetylhydrolase IB subunit alpha |
| Q9Y6K9 | IKBKG | NF-kappa-B essential modulator |
| Q09472 | EP300 | Histone acetyltransferase p300 |
| P40337 | VHL | von Hippel-Lindau disease tumor suppressor |
| O14678 | ABCD4 | ATP-binding cassette sub-family D member 4 |
| P01019 | AGT | Angiotensinogen |
| P07437 | TUBB | Tubulin beta chain |
| P16885 | PLCG2 | 1-phosphatidylinositol 4,5-bisphosphate phosphodiesterase gamma-2 |
| Q15147 | PLCB4 | 1-phosphatidylinositol 4,5-bisphosphate phosphodiesterase beta-4 |
| P30153 | PPP2R1A | Serine/threonine-protein phosphatase 2A 65 kDa regulatory subunit A alpha isoform |
| P50052 | AGTR2 | Type-2 angiotensin II receptor |
| Q13485 | SMAD4 | Mothers against decapentaplegic homolog 4, MAD homolog 4 |
| Q04656 | ATP7A | Copper-transporting ATPase 1 |
| P08754 | GNAI3 | Guanine nucleotide-binding protein G(k) subunit alpha |
| P78527 | PRKDC | DNA-dependent protein kinase catalytic subunit |
| P35222 | CTNNB1 | Catenin beta-1 |
| Q9UQB3 | CTNND2 | Catenin delta-2 |
| O60674 | JAK2 | Tyrosine-protein kinase JAK2 |
| O43683 | BUB1 | Mitotic checkpoint serine/threonine-protein kinase BUB1 |
| Q92834 | RPGR | X-linked retinitis pigmentosa GTPase regulator |
| P30556 | AGTR1 | Type-1 angiotensin II receptor |
| P60709 | ACTB | Actin, cytoplasmic 1 |
| P56381 | ATP5E | ATP synthase subunit epsilon, mitochondrial |
| P01111 | NRAS | GTPase Nras |
| P22681 | CBL | E3 ubiquitin-protein ligase CBL |
| P31751 | AKT2 | RAC-beta serine/threonine-protein kinase |
| P46783 | RPS10 | 40S ribosomal protein S10 |
| P51690 | ARSE | Arylsulfatase E |
| Q71U36 | TUBA1A | Tubulin alpha-1A chain |
| P09471 | GNAO1 | Guanine nucleotide-binding protein G(o) subunit alpha |
| P62273 | RPS29 | 40S ribosomal protein S29 |
| P60484 | PTEN | Phosphatidylinositol 3,4,5-trisphosphate 3-phosphatase and dual-specificity protein phosphatase PTEN |
| Q07889 | SOS1 | Son of sevenless homolog 1 |
| P60866 | RPS20 | 40S ribosomal protein S20 |
| P62081 | RPS7 | 40S ribosomal protein S7 |
| P19447 | ERCC3 | General transcription and DNA repair factor IIH helicase subunit XPB |
| O00203 | AP3B1 | AP-3 complex subunit beta-1 |
| Q9H9B1 | EHMT1 | Histone-lysine N-methyltransferase EHMT1 |
| Q9UQE7 | SMC3 | Structural maintenance of chromosomes protein 3 |
| O43924 | PDE6D | Retinal rod rhodopsin-sensitive cGMP 3',5'-cyclic phosphodiesterase subunit delta |
| P18074 | ERCC2 | General transcription and DNA repair factor IIH helicase subunit XPD |
| P11802 | CDK4 | Cyclin-dependent kinase 4 |
| P39019 | RPS19 | 40S ribosomal protein S19 |
| P21333 | FLNA | Filamin-A |
| P62857 | RPS28 | 40S ribosomal protein S28 |
| Q9NXR1 | NDE1 | Nuclear distribution protein nudE homolog 1 |
| P62847 | RPS24 | 40S ribosomal protein S24 |
| P62913 | RPL11 | 60S ribosomal protein L11 |
| O75643 | SNRNP200 | U5 small nuclear ribonucleoprotein 200 kDa helicase |
| P46777 | RPL5 | 60S ribosomal protein L5 |
| P19438 | TNFRSF1A | Tumor necrosis factor receptor superfamily member 1A |
| Q9UBN7 | HDAC6 | Histone deacetylase 6 |
| P09619 | PDGFRB | Platelet-derived growth factor receptor beta |
| O95613 | PCNT | Pericentrin |
| P18077 | RPL35A | 60S ribosomal protein L35a |
| P62854 | RPS26 | 40S ribosomal protein S26 |
| Q9NZJ5 | EIF2AK3 | Eukaryotic translation initiation factor 2-alpha kinase 3 |
| P35913 | PDE6B | Rod cGMP-specific 3',5'-cyclic phosphodiesterase subunit beta |
| Q14204 | DYNC1H1 | Cytoplasmic dynein 1 heavy chain 1 |
| P08708 | RPS17 | 40S ribosomal protein S17 |
| P15882 | CHN1 | N-chimaerin |
| P28340 | POLD1 | DNA polymerase delta catalytic subunit |
| P51955 | NEK2 | Serine/threonine-protein kinase Nek2 |
| P62380 | TBP | TATA box-binding protein-like protein 1 |
| P51571 | SSR4 | Translocon-associated protein subunit delta |
| P16499 | PDE6A | Rod cGMP-specific 3',5'-cyclic phosphodiesterase subunit alpha |
| O75914 | PAK3 | Serine/threonine-protein kinase PAK 3 |
| Q13415 | ORC1 | Origin recognition complex subunit 1 |
| P06850 | CRH | Corticoliberin |
| Q15029 | EFTUD2 | 116 kDa U5 small nuclear ribonucleoprotein component |
| P12272 | PTHLH | Parathyroid hormone-related protein |
| Q14683 | SMC1A | Structural maintenance of chromosomes protein 1A |
| P57078 | RIPK4 | Receptor-interacting serine/threonine-protein kinase 4 |
| O14746 | TERT | Telomerase reverse transcriptase |
| Q08499 | PDE4D | cAMP-specific 3',5'-cyclic phosphodiesterase 4D |
| Q9HC77 | CENPJ | Centromere protein J |
| P50148 | GNAQ | Guanine nucleotide-binding protein G(q) subunit alpha |
| P46109 | CRKL | Crk-like protein |
| P33681 | CD80 | T-lymphocyte activation antigen CD8 |
| O00444 | PLK4 | Serine/threonine-protein kinase PLK4 |
| O75665 | OFD1 | Oral-facial-digital syndrome 1 protein |
| P39656 | DDOST | Dolichyl-diphosphooligosaccharide--protein glycosyltransferase 48 kDa subunit |
| Q96SN8 | CDK5RAP2 | CDK5 regulatory subunit-associated protein 2 |
| Q15427 | SF3B4 | Splicing factor 3B subunit 4 |
| P18545 | PDE6G | Retinal rod rhodopsin-sensitive cGMP 3',5'-cyclic phosphodiesterase subunit gamma |
| Q86SQ7 | SDCCAG8 | Serologically defined colon cancer antigen 8 |
| Q9UGJ1 | TUBGCP4 | Gamma-tubulin complex component 4 |
| Q01968 | OCRL | Inositol polyphosphate 5-phosphatase OCRL-1 |
| Q03135 | CAV1 | Caveolin-1 |
| Q9Y5S9 | RBM8A | RNA-binding protein 8A |
| P23945 | FSHR | Follicle-stimulating hormone receptor |
| Q15738 | NSDHL | terol-4-alpha-carboxylate 3-dehydrogenase, decarboxylating |
| O14936 | CASK | Peripheral plasma membrane protein CASK |
| Q13627 | DYRK1A | Dual specificity tyrosine-phosphorylation-regulated kinase 1A |
| P16234 | PDGFRA | Platelet-derived growth factor receptor alpha |
| O15078 | CEP290 | Centrosomal protein of 290 kDa |
| Q96MT8 | CEP63 | Centrosomal protein of 63 kDa |
| Q8TAM2 | TTC8 | Tetratricopeptide repeat protein 8 |
| P35250 | RFC2 | Replication factor C subunit 2 |
| Q07890 | SOS2 | Son of sevenless homolog 2 |
| P25054 | APC | Adenomatous polyposis coli protein |
| Q8TB36 | GDAP1 | Ganglioside-induced differentiation-associated protein 1 |
| P63267 | ACTG2 | Actin, gamma-enteric smooth muscle |
| P14678 | SNRPB | Small nuclear ribonucleoprotein-associated proteins B and B' |
| O43929 | ORC4 | Origin recognition complex subunit 4 |
| Q99698 | LYST | Lysosomal-trafficking regulator |
| P28715 | ERCC5 | DNA repair protein complementing XP-G cells |
| P01127 | PDGFB | Platelet-derived growth factor subunit B |
| P35557 | GCK | Glucokinase |
| P51648 | ALDH3A2 | Fatty aldehyde dehydrogenase |
| O43172 | PRPF4 | U4/U6 small nuclear ribonucleoprotein Prp4 |
| Q03431 | PTH1R | Parathyroid hormone/parathyroid hormone-related peptide receptor |
| P07992 | ERCC1 | DNA excision repair protein ERCC-1 |
| Q00534 | CDK6 | Cyclin-dependent kinase 6 |
| O95602 | POLR1A | DNA-directed RNA polymerase I subunit RPA1 |
| P33991 | MCM4 | DNA replication licensing factor MCM4 |
| Q92989 | CLP1 | Polyribonucleotide 5'-hydroxyl-kinase Clp1 |
| P27986 | PIK3R1 | Phosphatidylinositol 3-kinase regulatory subunit alpha |
| P31749 | AKT1 | RAC-alpha serine/threonine-protein kinase |
| P15291 | B4GALT1 | Beta-1,4-galactosyltransferase 1 |
| P46531 | NOTCH1 | Neurogenic locus notch homolog protein 1 |
| P28069 | POU1F1 | Pituitary-specific positive transcription factor 1 |
| P27539 | GDF1 | Embryonic growth/differentiation factor 1 |
| O94766 | B3GAT3 | Galactosylgalactosylxylosylprotein 3-beta-glucuronosyltransferase 3 |
| P63261 | ACTG1 | Actin, cytoplasmic 2 |
| P30279 | CCND2 | G1/S-specific cyclin-D2 |
| Q14232 | EIF2B1 | Translation initiation factor eIF-2B subunit alpha |
| Q9BZI7 | UPF3B | Regulator of nonsense transcripts 3B |
| Q99714 | HSD17B10 | 3-hydroxyacyl-CoA dehydrogenase type-2 |
| Q08426 | EHHADH | Peroxisomal bifunctional enzyme |
| O15111 | CHUK | Inhibitor of nuclear factor kappa-B kinase subunit alpha, I-kappa-B kinase alpha |
| P45954 | ACADSB | Short/branched chain specific acyl-CoA dehydrogenase, mitochondrial |
| P21281 | ATP6V1B2 | V-type proton ATPase subunit B, brain isoform |
| P51532 | SMARCA4 | Transcription activator BRG1 |
| P52701 | MSH6 | DNA mismatch repair protein Msh6 |
| Q9BXC9 | BBS2 | Bardet-Biedl syndrome 2 protein |
| P51587 | BRCA2 | Breast cancer type 2 susceptibility protein |
| Q15052 | ARHGEF6 | Rho guanine nucleotide exchange factor 6 |
| P01133 | EGF | Pro-epidermal growth factor |
| Q6P2Q9 | PRPF8 | Pre-mRNA-processing-splicing factor 8 |
| O94906 | PRPF6 | Pre-mRNA-processing factor 6 |
| Q05516 | ZBTB16 | Zinc finger and BTB domain-containing protein 16 |
| P23025 | XPA | DNA repair protein complementing XP-A cells |
| Q9Y297 | BTRC | F-box/WD repeat-containing protein 1A |
| O43684 | BUB3 | Mitotic checkpoint protein BUB3 |
| Q9NX14 | NDUFB11 | NADH dehydrogenase [ubiquinone] 1 beta subcomplex subunit 11, mitochondrial |
| P28482 | MAPK1 | Mitogen-activated protein kinase 1 |
| P62258 | YWHAE | 14-3-3 protein epsilon |
| Q06124 | PTPN11 | Tyrosine-protein phosphatase non-receptor type 11 |
| Q05086 | UBE3A | Ubiquitin-protein ligase E3A |
| P49768 | PSEN1 | Presenilin-1 |
| P10644 | PRKAR1A | cAMP-dependent protein kinase type I-alpha regulatory subunit |
| P30566 | ADSL | Adenylosuccinate lyase |
| O14521 | SDHD | Succinate dehydrogenase [ubiquinone] cytochrome b small subunit, mitochondrial |
| P06213 | INSR | Insulin receptor |
| P49770 | EIF2B2 | Translation initiation factor eIF-2B subunit beta |
| P10253 | GAA | Lysosomal alpha-glucosidase |
| P01116 | KRAS | GTPase Kras |
| O00459 | PIK3R2 | Phosphatidylinositol 3-kinase regulatory subunit beta |
| Q15283 | RASA2 | Ras GTPase-activating protein 2 |
| P11511 | CYP19A1 | Aromatase |
| P05549 | TFAP2A | Transcription factor AP-2-alpha |
| P49454 | CENPF | Centromere protein F |
| P30968 | GNRHR | Gonadotropin-releasing hormone receptor |
| Q03468 | ERCC6 | DNA excision repair protein ERCC-6 |
| P43246 | MSH2 | DNA mismatch repair protein Msh2 |
| P01308 | INS | Insulin |
| P25705 | ATP5A1 | ATP synthase subunit alpha, mitochondrial |
| P23258 | TUBG1 | Tubulin gamma-1 chain |
| P63092 | GNAS | Guanine nucleotide-binding protein G(s) subunit alpha isoforms short |
| O60566 | BUB1B | Mitotic checkpoint serine/threonine-protein kinase BUB1 beta |
| P28328 | PEX2 | Peroxisome biogenesis factor 2 |
| Q01433 | AMPD2 | AMP deaminase 2 |
| Q9UPM8 | AP4E1 | AP-4 complex subunit epsilon-1 |
| P48643 | CCT5 | T-complex protein 1 subunit epsilon |
| Q13428 | TCOF1 | Treacle protein |
| O43272 | PRODH | Proline dehydrogenase 1, mitochondrial |
| P04792 | HSPB1 | Heat shock protein beta-1 |
| Q9NR50 | EIF2B3 | Translation initiation factor eIF-2B subunit gamma |
| Q01844 | EWSR1 | RNA-binding protein EWS |
| Q13586 | STIM1 | Stromal interaction molecule 1 |
| P01100 | FOS | Proto-oncogene c-Fos |
| P09622 | DLD | Dihydrolipoyl dehydrogenase, mitochondrial |
| Q92947 | GCDH | Glutaryl-CoA dehydrogenase, mitochondrial |
| P22695 | UQCRC2 | Cytochrome b-c1 complex subunit 2, mitochondrial |
| P23786 | CPT2 | Carnitine O-palmitoyltransferase 2, mitochondrial |
| P31150 | GDI1 | Rab GDP dissociation inhibitor alpha |
| P02545 | LMNA | Prelamin-A/C |
| P53779 | MAPK10 | Mitogen-activated protein kinase 10 |
| P40692 | MLH1 | DNA mismatch repair protein Mlh1 |
| P56524 | HDAC4 | Histone deacetylase 4 |
| P10721 | KIT | Mast/stem cell growth factor receptor Kit |
| Q99741 | CDC6 | Cell division control protein 6 homolog |
| P07196 | NEFL | Neurofilament light polypeptide |
| P49815 | TSC2 | Tuberin |
| O00238 | BMPR1B | Bone morphogenetic protein receptor type-1B |
| P54253 | ATXN1 | Ataxin-1 |
| P20823 | HNF1A | Hepatocyte nuclear factor 1-alpha |
| P37231 | PPARG | Peroxisome proliferator-activated receptor gamma |
| O60706 | ABCC9 | ATP-binding cassette sub-family C member 9 |
| P51812 | RPS6KA3 | Ribosomal protein S6 kinase alpha-3 |
| P08559 | PDHA1 | Pyruvate dehydrogenase E1 component subunit alpha, somatic form, mitochondrial |
| P16410 | CTLA4 | Cytotoxic T-lymphocyte protein 4 |
| P12643 | BMP2 | Bone morphogenetic protein 2 |
| O60890 | OPHN1 | Oligophrenin-1 |
| P23510 | TNFSF4 | Tumor necrosis factor ligand superfamily member 4 |
| P12074 | COX6A1 | Cytochrome c oxidase subunit 6A1 |
| O15287 | FANCG | Fanconi anemia group G protein |
| P07949 | RET | Proto-oncogene tyrosine-protein kinase receptor Ret |
| Q13950 | RUNX2 | Runt-related transcription factor 2 |
| P22607 | FGFR3 | Fibroblast growth factor receptor 3 |
| O75695 | RP2 | Protein XRP2 |
| Q12824 | SMARCB1 | SWI/SNF-related matrix-associated actin-dependent regulator of chromatin subfamily B member 1 |
| P21359 | NF1 | Neurofibromin |
| P08069 | IGF1R | Insulin-like growth factor 1 receptor |
| Q14526 | HIC1 | Hypermethylated in cancer 1 protein |
| P21397 | MAOA | Amine oxidase [flavin-containing] A |
| P53667 | LIMK1 | LIM domain kinase 1 |
| Q13535 | ATR | Serine/threonine-protein kinase ATR |
| P98174 | FGD1 | FYVE, RhoGEF and PH domain-containing protein 1 |
| P37173 | TGFBR2 | TGF-beta receptor type-2 |
| Q04721 | NOTCH2 | Neurogenic locus notch homolog protein 2 |
| P02452 | COL1A1 | Collagen alpha-1(I) chain |
| P11362 | FGFR1 | Fibroblast growth factor receptor 1 |
| P12644 | BMP4 | Bone morphogenetic protein 4 |
| Q14738 | PPP2R5D | Serine/threonine-protein phosphatase 2A 56 kDa regulatory subunit delta isoform |
| O14640 | DVL1 | Segment polarity protein dishevelled homolog DVL-1 |
| P10636 | MAPT | Microtubule-associated protein tau |
| P07237 | P4HB | Protein disulfide-isomerase |
| Q13224 | GRIN2B | Glutamate receptor ionotropic |
| O60488 | ACSL4 | Long-chain-fatty-acid--CoA ligase 4 |
| ACOX1 | ACOX1 | Peroxisomal acyl-coenzyme A oxidase 1 |
| P55072 | VCP | Transitional endoplasmic reticulum ATPase |
| Q09428 | ABCC8 | ATP-binding cassette sub-family C member 8 |
| O14788 | TNFSF11 | Tumor necrosis factor ligand superfamily member 11 |
| Q03393 | PTS | 6-pyruvoyl tetrahydrobiopterin synthase |
| P51451 | BLK | Tyrosine-protein kinase Blk |
| Q05639 | EEF1A2 | Elongation factor 1-alpha 2 |
| P52333 | JAK3 | Tyrosine-protein kinase JAK3 |
| Q8IY92 | SLX4 | Structure-specific endonuclease subunit SLX4 |
| O75369 | FLNB | Filamin-B |
| Q00535 | CDK5 | Cyclin-dependent-like kinase 5 |
| Q15831 | STK11 | Serine/threonine-protein kinase STK11 |
| Q9UHN1 | POLG2 | DNA polymerase subunit gamma-2 |
| P42772 | CDKN2B | Cyclin-dependent kinase 4 inhibitor B |
| Q13285 | NR5A1 | Steroidogenic factor 1 |
| O60333 | KIF1B | Kinesin-like protein KIF1B |
| O15265 | ATXN7 | Ataxin-7 |
| P29973 | CNGA1 | cGMP-gated cation channel alpha-1 |
| Q16620 | NTRK2 | BDNF/NT-3 growth factors receptor |
| Q14331 | FRG1 | Protein FRG1 |
| Q8NFJ9 | BBS1 | Bardet-Biedl syndrome 1 protein |
| Q99643 | SDHC | Succinate dehydrogenase cytochrome b560 subunit |
| O00571 | DDX3X | ATP-dependent RNA helicase DDX3X |
| P55075 | FGF8 | Fibroblast growth factor 8 |
| Q8IXJ9 | ASXL1 | Putative Polycomb group protein ASXL1 |
| P33897 | ABCD1 | ATP-binding cassette sub-family D member 1 |
| Q9BVA1 | TUBB2B | Tubulin beta-2B chain |
| P63162 | SNRPN | Small nuclear ribonucleoprotein-associated protein N |
| O00429 | DNM1L | Dynamin-1-like protein |
| P11055 | MYH3 | Myosin-3 |
| P13804 | ETFA | Electron transfer flavoprotein subunit alpha |
| O00548 | DLL1 | Delta-like protein 1 |
| P41235 | HNF4A | Hepatocyte nuclear factor 4-alpha |
| P14923 | JUP | Junction plakoglobin |
| P04610 | TAT | Transactivating regulatory protein |
| Q9BRB3 | PIGQ | Phosphatidylinositol N-acetylglucosaminyltransferase subunit Q |
| P39905 | GDNF | Glial cell line-derived neurotrophic factor |
| P15924 | DSP | Desmoplakin |
| P21802 | FGFR2 | Fibroblast growth factor receptor 2 |
| Q13501 | SQSTM1 | Sequestosome-1 |
| O00294 | TULP1 | Tubby-related protein 1 |
| P23284 | PPIB | Peptidyl-prolyl cis-trans isomerase B |
| Q99967 | CITED2 | Cbp/p300-interacting transactivator 2 |
| P23760 | PAX3 | Paired box protein Pax-3 |
| P46100 | ATRX | Transcriptional regulator ATRX |
| P56192 | MARS | Methionine--tRNA ligase, cytoplasmic |
| P08123 | COL1A2 | Collagen alpha-2(I) chain |
| O15360 | FANCA | Fanconi anemia group A protein |
| Q99986 | VRK1 | Serine/threonine-protein kinase VRK1 |
| P24158 | PRTN3 | Myeloblastin |
| Q14563 | SEMA3A | Semaphorin-3A |
| Q9P2R7 | SUCLA2 | Succinate--CoA ligase [ADP-forming] subunit beta, mitochondrial |
| P51610 | HCFC1 | Host cell factor 1 |
| Q96S42 | NODAL | Nodal homolog |
| Q99453 | PHOX2B | Paired mesoderm homeobox protein 2B |
| P15884 | TCF4 | Transcription factor 4 |
| Q5JTC6 | AMER1 | APC membrane recruitment protein 1 |
| Q13591 | SEMA5A | Semaphorin-5A |
| P41159 | LEP | Leptin |
| Q05193 | DNM1 | Dynamin-1 |
| ABCA4 | ABCA4 | Retinal-specific ATP-binding cassette transporter |
| P48436 | SOX9 | Transcription factor SOX-9 |
| Q01959 | SLC6A3 | Sodium-dependent dopamine transporter |
| Q99608 | NDN | Necdin |
| Q13562 | NEUROD1 | Neurogenic differentiation factor 1 |
| P11161 | EGR2 | E3 SUMO-protein ligase EGR2 |
| Q92481 | TFAP2B | Transcription factor AP-2-beta |
| P04156 | PRNP | Major prion protein |
| Q92994 | BRF1 | Transcription factor IIIB 90 kDa subunit |
| P50542 | PEX5 | Peroxisomal targeting signal 1 receptor |
| P47897 | QARS | Glutamine--tRNA ligase |
| Q9Y6B7 | AP4B1 | AP-4 complex subunit beta-1 |
| P13385 | TDGF1 | Teratocarcinoma-derived growth factor 1 |
| P15976 | GATA1 | Erythroid transcription factor |
| Q13635 | PTCH1 | Protein patched homolog 1 |
| P26367 | PAX6 | Paired box protein Pax-6 |
